# Technical skill assessment in minimally invasive surgery using artificial intelligence: a systematic review

**DOI:** 10.1007/s00464-023-10335-z

**Published:** 2023-08-16

**Authors:** Romina Pedrett, Pietro Mascagni, Guido Beldi, Nicolas Padoy, Joël L. Lavanchy

**Affiliations:** 1 Department of Visceral Surgery and Medicine, Inselspital, Bern University Hospital, University of Bern, Bern, Switzerland; 2 IHU Strasbourg, Strasbourg, France; 3 Fondazione Policlinico Universitario A. Gemelli IRCCS, Rome, Italy; 4 ICube, CNRS, University of Strasbourg, Strasbourg, France; 5 University Digestive Health Care Center Basel – Clarunis, PO Box, 4002 Basel, Switzerland

**Keywords:** Technical skill assessment, Surgical skill assessment, Artificial intelligence, Minimally invasive surgery, Surgical data science

## Abstract

**Background:**

Technical skill assessment in surgery relies on expert opinion. Therefore, it is time-consuming, costly, and often lacks objectivity. Analysis of intraoperative data by artificial intelligence (AI) has the potential for automated technical skill assessment. The aim of this systematic review was to analyze the performance, external validity, and generalizability of AI models for technical skill assessment in minimally invasive surgery.

**Methods:**

A systematic search of Medline, Embase, Web of Science, and IEEE Xplore was performed to identify original articles reporting the use of AI in the assessment of technical skill in minimally invasive surgery. Risk of bias (RoB) and quality of the included studies were analyzed according to Quality Assessment of Diagnostic Accuracy Studies criteria and the modified Joanna Briggs Institute checklists, respectively. Findings were reported according to the Preferred Reporting Items for Systematic Reviews and Meta-Analyses statement.

**Results:**

In total, 1958 articles were identified, 50 articles met eligibility criteria and were analyzed. Motion data extracted from surgical videos (*n* = 25) or kinematic data from robotic systems or sensors (*n* = 22) were the most frequent input data for AI. Most studies used deep learning (*n* = 34) and predicted technical skills using an ordinal assessment scale (*n* = 36) with good accuracies in simulated settings. However, all proposed models were in development stage, only 4 studies were externally validated and 8 showed a low RoB.

**Conclusion:**

AI showed good performance in technical skill assessment in minimally invasive surgery. However, models often lacked external validity and generalizability. Therefore, models should be benchmarked using predefined performance metrics and tested in clinical implementation studies.

**Supplementary Information:**

The online version contains supplementary material available at 10.1007/s00464-023-10335-z.

The assessment of technical skill is of major importance in surgical education and quality improvement programs given the association of technical skills with clinical outcomes [1–4]. This correlation has been demonstrated among others in bariatric [1], upper gastrointestinal [2], and colorectal surgery [3, 4]. In addition, data from the American Colleges of Surgeons National Surgical Quality Improvement Program revealed that surgeon’s technical skills as assessed by peers during right hemicolectomy are correlated with outcomes in colorectal as well as in non-colorectal surgeries performed by the same surgeon [3], showing the overarching impact of technical skills on surgical outcomes.

In surgical education, technical skills of trainees are often assessed by staff surgeons through direct observations in the operating room. These instantaneous assessments by supervisors are frequently unstructured and might only be snapshots of the actual technical performance of a trainee. Furthermore, they often lack objectivity due to peer review bias [5]. Aiming to improve the objectivity and construct validity of technical skill assessment, video-based assessment has been introduced [6]. Video-based assessment allows for retrospective review of full-length procedures or critical phases of an intervention by one or multiple experts. Despite the improvement of technical skill assessment by video-based assessment, it is still limited by the need for manual review of procedures by experts. Therefore, technical skill assessment is time-consuming, costly, and not scalable.

Automation of video-based assessment using artificial intelligence (AI) could lead to affordable, objective, and consistent technical skill assessment in real-time.

Despite the great potential of AI in technical skill assessment, it remains uncertain how accurate, valid, and generalizable AI models are to date. Therefore, the aim of this systematic review was to analyze the performance, external validity, and generalizability of AI models for technical skill assessment in minimally invasive surgery.

## Methods

This systematic review is reported in accordance with the PRISMA (Preferred Reporting Items for Systematic Reviews and Meta-Analyses) [7] guidelines and was prospectively registered at PROSPERO (2021 CRD42021267714). The PRISMA checklist can be found in the Supplementary (Table S1).

### Literature search

A systematic literature search of the databases Medline/Ovid, Embase/Ovid, Web of Science, and IEEE Explore was conducted on August 25th, 2021. The first three databases account for biomedical literature and IEEE Explore for technical literature. A librarian at the University Library, University of Bern performed the literature search combining the following terms using Boolean operators: (1) Minimally invasive surgery including laparoscopic, or robotic surgery, and box model trainer. (2) AI including machine learning (ML), supervised learning, unsupervised learning, computer vision, and convolutional neural networks. (3) Technical skill assessment including surgical skill assessment, surgical performance assessment, and task performance analysis. The full-text search terms are shown in the Supplementary (Table S2). The literature search was re-run prior to final analysis on February 25th, 2022 and May 31st, 2023.

### Eligibility criteria

Studies presenting original research on AI applications for technical skills assessment in minimally invasive surgery including box model trainers published within the last 5 years (08/2016-08/2021, updated 02/2022 & 05/2023) in English language were included. Review articles, conference abstracts, comments, and letters to the editor were excluded.

Any form of quantitative or qualitative evaluation of manual surgical performance was considered a technical skill assessment.

### Study selection

Before screening, the identified records were automatically deduplicated using the reference manager program Endnote™ (Clarivate Analytics). After removal of the duplicates, two authors (R.P. & J.L.L.) independently screened the titles and abstracts of the identified records for inclusion using the web-tool Rayyan (https://www.rayyan.ai) [8]. Disagreement of the two authors regarding study selection was settled in joint discussion. Of all included records the full-text articles were acquired. Articles not fulfilling the inclusion criteria after full-text screening were excluded.

### Data extraction

Besides bibliographic data (title, author, publication year, journal name), the study population, the setting (laparoscopic/robotic simulation or surgery), the task assessed (e.g., peg transfer, cutting, knot-tying), the data input (motion data from video recordings, kinematic data from robotic systems or sensors), the dataset used (a dataset is a defined collection of data either especially collected for the aim of the study or reused from previous studies), the assessment scale (ordinal scale vs. interval scale), the AI models used [ML or deep learning (DL)], the performance and the maturity level (development, validation, implementation) of AI models were extracted from the included studies. Missing or incomplete data was not imputed.

### Performance metrics

The performance of AI models in technical skill assessment can be measured as accuracy, precision, recall, F1-score, and Area Under the Curve of Receiver Operator Characteristic (AUC-ROC). This paragraph gives a short definition of the used performance metrics. Accuracy is the proportion of correct predictions among the total number of observations. Precision is the proportion of true positive predictions among all (true and false) positive predictions and referred to as the positive predictive value. Recall is the proportion of true positive predictions among all relevant observations (true positives and false negatives) and referred to as sensitivity. F1-score is the harmonic mean of precision and recall and is a measure of model performance. A ROC curve plots the true positive against the false positive predictions at various thresholds and the AUC describes performance of the model to distinguish true positive from false positive predictions.

### Risk of bias and quality assessment

The risk of bias (RoB) of the included studies was assessed using the modified version of Quality Assessment of Diagnostic Accuracy Studies (QUADAS-2) criteria [9]. This tool is commonly used for RoB evaluation in quality assessment studies. The quality of studies was evaluated using the modified Joanna Briggs Institute critical appraisal checklist for cross-sectional research in ML as used in [10, 11].

## Results

**Fig. 1:**
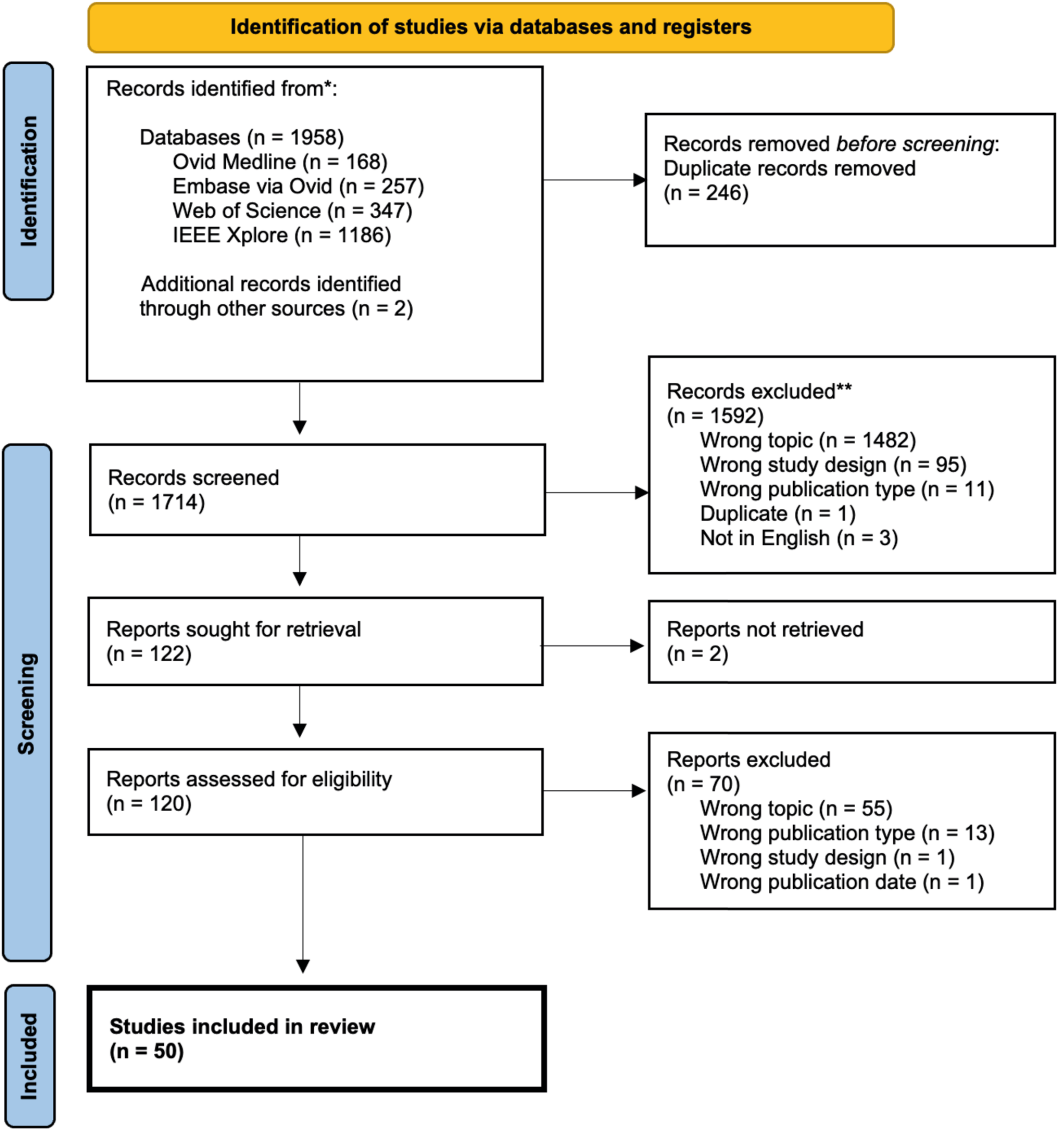
PRISMA flow diagram of the study selection process (from PRISMA Statement 2020) [7]

The literature search retrieved a total of 1958 studies. After removing all duplicates, the remaining 1714 studies were screened by title and abstract. Thereafter, 120 studies remained, of which 70 were excluded after full-text screening. In summary, 50 studies [12–61] met the eligibility criteria and thus were included into this systematic review (Fig. 1). Two of the 50 studies [34, 61] included in this review were found to match the inclusion criteria during the process of full-text screening and were thus included through cross-referencing. Six studies [21, 29, 37, 45, 55, 58] were obtained during the re-run prior to final analysis six months after the initial literature search and 13 [13, 17, 34, 36, 38, 42, 48, 50, 52–54, 56, 61] during the second update on May 31st, 2023. Table 1 gives an overview of the 50 studies included in this systematic review (for full information extracted see Supplementary Table S3).

**Table 1:** Information summary of all studies included in this review

Author	Year	Population	Setting	Tasks	Input data	Dataset	Assessment	AI model	Accuracy	Maturity level
Alonso-Silverio et al. [12]	2018	20	LS	PC	VR	Private	Binary (experienced, non-experienced)	DL	0.94	Dev
Anastasiou et al. [13]	2023	8	RS	SU, NP, KT	VR	JIGSAWS	Modified OSATS score	DL	na	Dev
Anh et al. [14]	2020	8	RS	SU, NP, KT	KD (dV)	JIGSAWS	N, I, E	DL	0.97	Dev
Baghdadi et al. [15]	2018	na	Lap	Pelvic lymph node dissection	VR	Private	PLACE score	ML	0.83	Dev
Benmansour et al. [16]	2018	6	RS	SU, NP, KT	KD (dV)	JIGSAWS	Custom score	DL	na	Dev
Benmansour et al. [17]	2023	8	RS	SU, NP, KT	KD (dV)	JIGSAWS	OSATS score	DL	na	Dev
Brown et al. [18]	2017	38	RS	PT	KD (dV)	Private	GEARS score (1–5: exact rating)	ML	0.75	Dev
Castro et al. [19]	2019	8	RS	SU, NP, KT	KD (dV)	JIGSAWS	N, I, E	DL	0.98	Dev
Fard et al. [20]	2017	8	RS	SU, KT	KD (dV)	JIGSAWS	Binary (N, E)	ML	0.9	Dev
Fathabadi et al. [21]	2021	na	LS	PC	VR	Private	Level A (excellent)—E (very bad)	DL	na	Dev
Fawaz et al. [22]	2019	8	RS	SU, NP, KT	KD (dV)	JIGSAWS	N, I, E	DL	1	Dev
Forestier et al. [23]	2018	8	RS	SU, NP, KT	KD (dV)	JIGSAWS	N, I, E	ML	0.96	Dev
French et al. [24]	2017	98	LS	PT, SU, PC	VR	Private	Binary (N, E)	ML	0.9	Dev
Funke et al. [25]	2019	8	RS	SU	VR	JIGSAWS	N, I, E	DL	1	Dev
Gao et al. [26]	2020	13	LS	PC	fNIRS data	Private	FLS score: pass/fail	DL	0.91	Dev
Islam et al. [27]	2016	52	LS	PT, SU, PC	VR	Private	Custom score	DL	na	Dev
Jin et al. [28]	2018	na	Lap	Lap cholecystectomy	VR	m2cai16-tools-location	Qualitative description	DL	na	Dev
Juarez-Villalobos et al. [29]	2021	8	RS	SU, NP, KT	KD (dV)	JIGSAWS	Binary (N, E)	ML	1	Dev
Keles et al. [30]	2021	33	LS	PT, threading	fNIRS data	Private	Binary (student vs. attending)	ML	~ 0.9	Dev
Kelly et al. [31]	2020	na	LS	PT, SU, PC, Clipping	VR	Private	Binary (N, E)	DL	0.97	Dev
Khalid et al. [32]	2020	8	RS	SU, NP, KT	VR	JIGSAWS	N, I, E	DL	0.77	Dev
Kitaguchi et al. [33]	2021	na	Lap	Lap colorectal surgery	VR	Private	ESSQS score	DL	0.75	Dev
Kiyasseh et al. [34]	2023	42	Rob	SU	VR	Private	Binary (low vs. high skill level)	ML	na	Dev
Kowalewski et al. [35]	2019	28	LS	SU, KT	KD (s)	Private	N, I, E	DL	0.7	Dev
Kuo et al. [36]	2022	10	LS	PT	VR, ETD	Private	N, I, E	ML, DL	0.83	Dev
Lajkó et al. [37]	2021	8	RS	SU, NP, KT	KD (dV)	JIGSAWS	Binary (N, E)	DL	0.84	Dev
Lam et al. [38]	2022	na	Lap	Lap gastric band insertion	VR	Private	Binary (trainee vs. expert)	DL	na	Dev
Lavanchy et al. [39]	2021	40	Lap	Lap cholecystectomy	VR	Private	Binary (good vs. poor)	ML, DL	0.87	Dev
							5-point Likert scale (± 1 point)		0.7	
Laverde et al. [40]	2018	7	LS	PT	KD (s)	Private	N, I, E	DL	na	Dev
Law et al. [41]	2017	12	Rob	Robotic prostatectomy	VR	Private	Binary (good vs. poor)	ML, DL	0.92	Dev
Lazar et al. [42]	2023	27	LS	PT	VR	Private	N, I, E	DL	na	Dev
Lee et al. [43]	2020	1/ na	RS, Rob	Robotic thyroid surgery/simulation	VR	Private	N, I, E	DL	0.83	Dev
Liu et al. [44]	2020	na	Lap	Lap gastrectomy	VR	Private	Modified OSATS score	DL	na	Dev
Liu et al. [45]	2021	8 / na	RS, Lap	SU, NP, KT / Lap surgery gastric cancer	VR	JIGSAWS	Modified OSATS score	ML	na	Dev
Lyman et al. [46]	2021	2	RS	SU of hepaticojejunostomy	KD (dV)	Private	Binary (N, I)	ML	0.89	Dev
Nguyen et al. [47]	2019	8	RS	SU, NP, KT	KD (dV)	JIGSAWS	N, I, E	DL	0.98	Dev
Oğul et al. [48]	2022	8 / 12	RS	SU, NP, KT / post and sleeve, pea on a peg and wire chaser	KD (dV)	JIGSAWS / ROSMA	Binary (JIGSAWS)	CNN	0.79	Dev
							Pairwise ranking (ROSMA)		0.75	
Oquendo et al. [49]	2018	32	LS	SU	KD (s)	Private	OSATS score (± 4 points)	ML	0.89	Dev
Pan et al. [50]	2023	8	RS	SU	VR	JIGSAWS	Binary	DL	0.92	Dev
							N, I, E		0.85	
Pérez-Escamirosa et al. [51]	2019	43	LS	PT, SU, PC	VR	Private	Binary (experienced vs. non-experienced)	ML	0.98	Dev
Sasaki et al. [52]	2022	na	Lap	Lap sigmoidectomy	VR	Private	N, I, E (based on blood pixels)	ML	na	Dev
Shafiei et al. [53]	2023	11	RS	bluntD, retraction, coldD, burnD	ETD	Private	N, I, E	ML	0.96	Dev
Soangra et al. [54]	2022	26	LS, RS	PT, KT	KD (s)	Private	N, I, E	ML	0.58	Dev
Soleymani et al. [55]	2021	8	RS	SU, NP, KT	VR	JIGSAWS	N, I, E	DL	0.97	Dev
Soleymani et al. [56]	2022	8	RS	SU, NP, KT	KD (dV)	JIGSAWS	Qualitative description	DL	na	Dev
Uemura et al. [57]	2018	67	LS	SU	KD (s)	Private	Binary (N, E)	DL	0.79	Dev
Wang Y. et al. [58]	2021	18	RS	SU	VR	Private	N, I, E	DL	0.83	Dev
							GEARS score (± 1 point)		0.86	
Wang Z. et al. [59]	2018	8	RS	SU, NP, KT	KD (dV)	JIGSAWS	N, I, E	DL	0.95	Dev
Wang Z. et al. [60]	2018	8	RS	SU, NP, KT	KD (dV)	JIGSAWS	N, I, E	DL	0.96	Dev
Zia et al. [61]	2018	8	RS	SU, NP, KT	KD (dV)	JIGSAWS	N, I, E	ML	na	Dev
							Modified OSATS score		na	

Of note, to ensure legibility the data provided in Table 1 is limited to accuracy metrics of the best performing model presented in each study. For full information extracted see Supplementary Material Table S3

*na* not available, *LS* laparoscopic simulator, *Lap* laparoscopic surgery, *RS* robotic simulator, *Rob* robotic surgery, *PC* pattern cutting, *SU* suturing, *NP* needle-passing, *KT* knot-tying, *PT* peg transfer, *bluntD* blunt dissection, *coldD* cold dissection (incl. cutting with scissors), *burnD* burn dissection, *VR* video recordings, *KD (dv)* kinematic data collected by Da Vinci systems, *fNIRS* functional near-infrared spectroscopy, *KD (s)* kinematic data collected by external sensors, *ETD* eye tracker data, *DL* deep learning, *ML* machine learning, *N* novice, *I* intermediate, *E* expert, *PLACE* Pelvic Lymphadenectomy Assessment and Completion Evaluation, *GEARS* Global Evaluative Assessments of Robotic Skills, *FLS* Fundamentals of Laparoscopic Surgery, *AICS* AI confidence score, *ESSQS* Endoscopic Surgical Skill Qualification System, *OSATS* Objective Structured Assessment of Technical Skills, *dev* development

### Settings and tasks

Most often, motion data from surgical videos or kinematic data from robotic systems or sensors were collected from simulators rather than during actual surgical procedures. The most common simulators used were robotic box models (*n* = 27, 54%) [13, 14, 16–20, 22, 23, 25, 29, 32, 37, 43, 45–48, 50, 53–56, 58–61]. Laparoscopic simulators were the second most common setting for data collection (*n* = 15, 30%) [12, 21, 24, 26, 27, 30, 31, 35, 36, 40, 42, 49, 51, 54, 57].

The most common tasks assessed were suturing (*n* = 31, 62%) [13, 14, 16, 17, 19, 20, 22–25, 27, 29, 31, 32, 34, 35, 37, 45–51, 55–61], knot-tying (*n* = 21, 42%) [13, 14, 16, 17, 19, 20, 22, 23, 29, 32, 35, 37, 45, 47, 48, 54–56, 59–61], and needle passing (*n* = 18, 36%) [13, 14, 16, 17, 19, 22, 23, 29, 32, 37, 45, 47, 48, 55, 56, 59–61]. Other tasks assessed were peg transfers (*n* = 10, 20%) [18, 24, 27, 30, 31, 36, 40, 42, 51, 54] and pattern cutting (*n* = 7) [12, 21, 24, 26, 27, 31, 51]. All these tasks are part of the Fundamentals of Laparoscopic Surgery program, a well-established training curriculum for laparoscopic surgery with proven construct validity [62, 63].

Eleven studies (22%) [15, 28, 33, 34, 38, 39, 41, 43–45, 52] used data of real surgical procedures. Eight [15, 28, 33, 38, 39, 44, 45, 52] of them using videos of laparoscopic surgeries as for example laparoscopic cholecystectomies [28, 39] or laparoscopic pelvic lymph node dissections [15]. Three studies [34, 41, 43] used video data obtained from robotic surgeries such as robotic prostatectomy [41] or robotic thyroid surgery [43]. The tasks assessed in surgical procedures ranged from entire interventions to specific steps (e.g., lymph node dissection [15], clip application [39]).

### Input data

Four different types of input data were used throughout the 50 studies: video data (*n* = 25, 50%) [12, 13, 15, 21, 24, 25, 27, 28, 31–34, 36, 38, 39, 41–45, 50–52, 55, 58], kinematic data (*n* = 22, 44%) [14, 16–20, 22, 23, 29, 35, 37, 40, 46–49, 54, 56, 57, 59–61], eye tracking data (*n* = 2) [36, 53], and functional near-infrared spectroscopy (fNIRS) data (*n* = 2) [26, 30]. Video recordings either from laparoscopic/robotic cameras or external cameras are used in 25 studies (50%). Kinematic data was obtained from DaVinci robotic systems (Intuitive Surgical Inc., CA, USA) in 17 studies (34%) [14, 16–20, 22, 23, 29, 37, 46–48, 56, 59–61] and from external sensors in five studies [35, 40, 49, 54, 57]. For example, electromyography sensors (Myo armband, Thalmic Labs, Ontario, CA) [35], optical sensors (Apple Watch, Apple, CA, USA) [40] or magnetic sensors attached to the instruments [49, 57] were used as external sensors to collect kinematic data. Two studies [26, 30] recorded fNIRS data from participants while they performed laparoscopic tasks. For example, Keles et al. [30] collected fNIRS data using a wireless, high density NIRS device, measuring functional brain activation of the prefrontal cortex. The NIRS device was adjacent to the surgeons’ foreheads while they performed different laparoscopic tasks. Another approach was the tracking of eye gaze data. For example, Kuo et al. [36] used the Pro Nano (Tobii Technology, Stockholm, Sweden) remote eye tracker to record gaze points during the tasks.

### Datasets and external validation

Publicly available datasets were used in 22 studies (44%) [13, 14, 16, 17, 19, 20, 22, 23, 25, 28, 29, 32, 37, 45, 47, 48, 50, 55, 56, 59–61]. Of those, the JIGSAWS (Johns Hopkins University and Intuitive Surgical, Inc. Gesture and Skill Assessment Working Set) [64] dataset was most frequently used (*n* = 21, 42%) [13, 14, 16, 17, 19, 20, 22, 23, 25, 29, 32, 37, 45, 47, 48, 50, 55, 56, 59–61]. It contains video and kinematic data together with human annotated skill ratings of eight surgeons performing three surgical tasks in five-fold repetition in a robotic box model trainer. Oğul et al. [48] used another, newly released publicly available dataset called Robotic Surgical Maneuvers (ROSMA) dataset [65]. This dataset recorded using the Da Vinci Research kit provides dynamic and kinematic data as well as a performance score calculated from time to completion and penalty points. One study [28] extended the publicly available m2cai16-tool dataset [66] with locations of surgical tools and published it as m2cai16-tools-localisation dataset. Though, most studies (*n* = 28, 56%) [12, 15, 18, 21, 24, 26, 27, 30, 31, 33–36, 38–44, 46, 49, 51–54, 57, 58] created private datasets, that were not publicly released. Most datasets (*n* = 46, 92%) [12–23, 25–32, 35–40, 42–61] were monocentric. However, four studies used a multicentric dataset: French, et al. [24] used a multi-institutional dataset from three centers, Kitagutchi, et al. [33] drew a sample form a national Japan Society of Endoscopic Surgeons database, Kiyasseh et al. [34] trained on data from one center and deployed the model to two other centers, and Law, et al. [41] used a part of a statewide national quality improvement database collected by the Michigan Urological Surgical Improvement Collaborative. Four of the 50 studies included [23, 34, 45, 47], reported external validation on a second independent dataset.

### Assessment

**Fig. 2:**
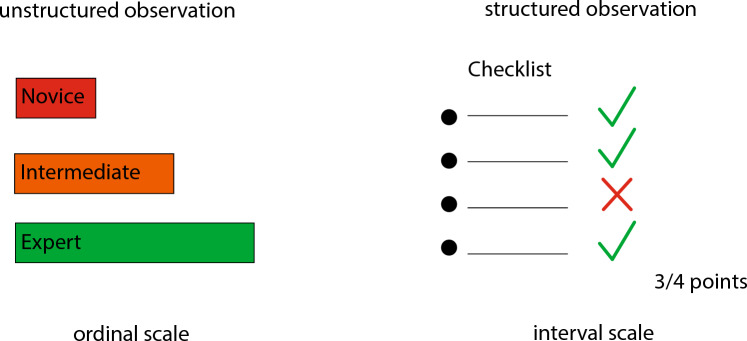
Human technical skill assessment in minimally invasive surgery

Technical surgical skills can be assessed using expert levels (ordinal scale) or proficiency scores (interval scale) (Fig. 2). In 36 of the studies (72%) an ordinal scale was applied [12, 14, 19–25, 29–32, 34, 35, 37–43, 46–48, 50–55, 57–61]. In 16 studies (32%) participants were categorized in two different skill levels [12, 20, 24, 29–31, 34, 37–39, 41, 46, 48, 50, 51, 57] and in 20 studies (40%) into three different expert levels (novice, intermediate, expert) [14, 19, 22, 23, 25, 32, 35, 36, 40, 43, 47, 50, 52–55, 58–61]. Twelve studies (24%) applied different proficiency scores: Pelvic Lymphadenectomy Assessment and Completion Evaluation (PLACE [67]), Fundamentals of Laparoscopic Surgery (FLS [68]), Endoscopic Surgical Skill Qualification System (ESSQS [69]), Objective Structured Assessment of Technical Skills (OSATS [70]), and Global Evaluative Assessment of Robotic Skills (GEARS [71]) [15–18, 26, 27, 33, 44, 45, 49, 58, 61].

### AI models

**Fig. 3:**
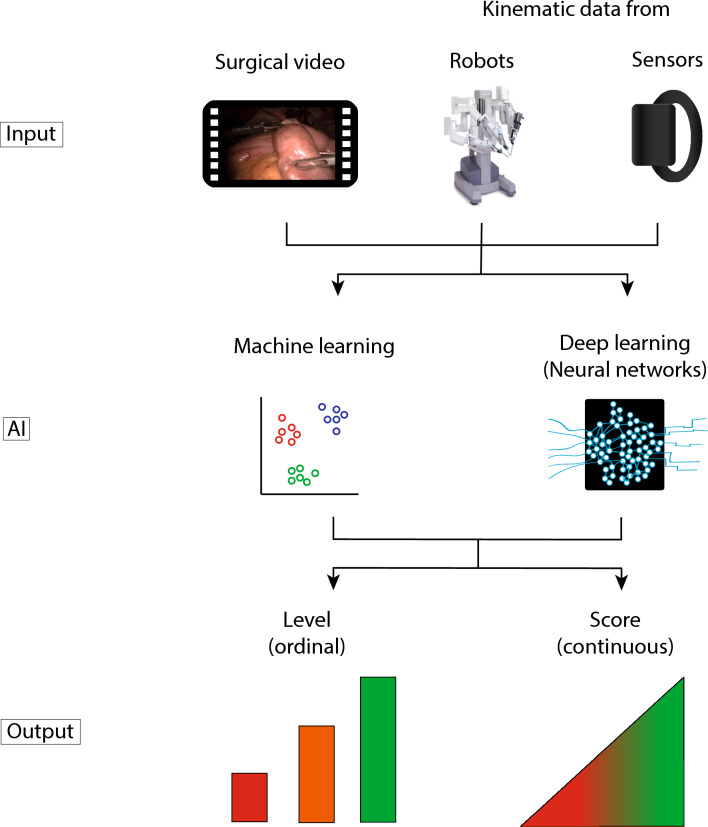
Automated technical skill assessment in minimally invasive surgery by artificial intelligence

All AI models in this review are either ML- or DL-based (Fig. 3). ML was applied in 19 studies (38%) [18, 20, 23, 24, 29, 30, 34–36, 39, 41, 45, 46, 49, 51–54, 61] and DL in 34 studies (68%) [12–14, 16, 17, 19, 21, 22, 25–28, 31–33, 35–44, 47, 48, 50, 55–60]. Three studies used a combination of ML and DL models [36, 39, 41].

### Performance

The most common performance metric reported in the studies included in this systematic review is accuracy (*n* = 35, 70%) [12, 14, 15, 18–20, 22–26, 29–33, 35–37, 39, 41, 43, 46–51, 53–55, 57–60]. Accuracies of the best performing models range between 0.58 and 1. Other performance metrics reported include F1-score (*n* = 9) [25, 29, 32, 40, 50, 51, 53, 54, 60], recall also known as sensitivity (*n* = 11, 22%) [12, 18, 25, 26, 32, 35, 50, 51, 53, 54, 60], specificity (*n* = 4) [14, 26, 44, 45], and AUC-ROC (*n* = 4) [14, 29, 44, 45]. Six studies [16, 21, 27, 28, 42, 56] did not report a performance metric at all.

### Risk of bias and quality assessment

**Fig. 4:**
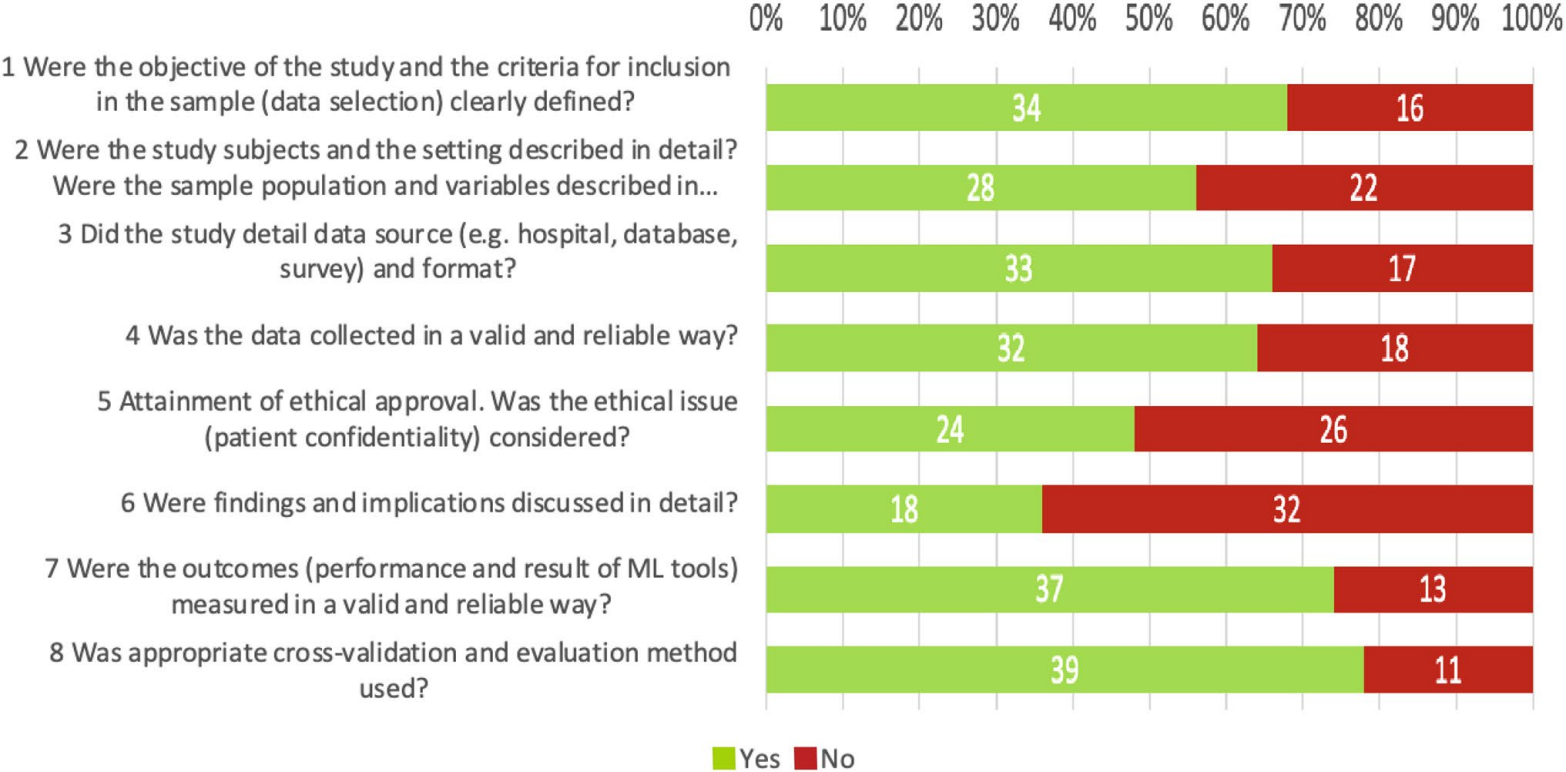
Quality assessment of the included studies. The numbers within the bars represent the respective number of studies

Eight of the included studies [18, 31, 33–35, 39, 49, 53] had an overall low probability of bias in the RoB assessment. The other studies had one (*n* = 15, 30%) [25, 26, 30, 32, 36, 40, 41, 44, 45, 50–52, 56, 59, 61], two (*n* = 13, 26%) [12, 13, 19, 22–24, 29, 42, 47, 48, 54, 55, 58], three (*n* = 9) [12, 13, 19, 22–24, 29, 42, 47, 48, 54, 55, 58], four (*n* = 4) [15, 17, 21, 46] or five criteria (*n* = 1) [16] at RoB. The full RoB assessment table is presented in the Supplementary (Table S4). The quality assessment of the included studies is displayed in Fig. 4. All proposed AI models were in a developmental preclinical stage of maturity, none was implemented in routine clinical use.

## Discussion

This systematic review of AI applications for technical skill assessment in minimally invasive surgery assessed the performance, external validity, and generalizability of 50 included studies. A large variety of task, settings, datasets, and AI models have been studied.

In general, technical skill assessment involves either classifying skill levels in ordinal scales (e.g., novice, intermediate and expert) through unstructured observations or assessing performance intervals using structured checklists (e.g., Objective Structured Assessment of Technical Skills (OSATS) [70], Global Evaluative Assessment of Robotic Skills (GEARS) [71]) (Fig. 2). OSATS for example evaluates technical skills in seven dimensions (respect for tissue, time and motion, instrument handling, knowledge of instruments, use of assistants, flow of operation and forward planning, and knowledge of specific procedure) assigning a 5-point Likert scale from 1 (low skill) to 5 (high skill) to every dimension. Thus, 35 points is the maximum OSATS score reflecting highest technical skills. The ideal automated skill assessment model would not just output a skill level or overall score, but rather multiple dimensions of skill to provide actionable feedback to trainees.

Two subfields of AI are particularly used to extract and analyze motion data from surgical videos or robotic systems to assess technical skill: ML and DL. ML can be defined as computer algorithms that learn distinct features iterating over data without explicit programming. DL designates computer algorithms that analyze unstructured data using neural networks (NN). NN are computer algorithms designed in analogy to the synaptic network of the human brain. The input data is processed through multiple interconnected layers of artificial neurons, each performing mathematical operations on the input data to predict an output. The predicted output is compared to the human labeled output to optimize the operations of the NN, which makes it a self-learning system. From an AI perspective technical skill assessment is a classification (prediction of expert levels) or a regression task (prediction of a score). Figure 3 illustrates how different input data types are processed by AI models to predict technical skills.

The generalizability of the studies included in this systematic review is limited due to several fundamental differences between them. Most studies (56%) used private datasets of different settings, tasks, and sizes. However, 21 studies (42%) included in this systematic review used JIGSAWS, a robotic simulator dataset and the most frequently used dataset in technical skill assessment. The use of simulators for technical skill assessment has advantages and disadvantages. On the one hand, simulators allow to control the experimental setting and enable reproducibility of studies. On the other hand, box model trainers simulate surgical tasks and have only a restricted degree of realism. In addition, simulators are well established in surgical training but have limited significance in the assessment of fully trained surgeons. The use of video recordings and motion data of actual surgeries as input data improves the construct validity of technical skill assessment models. However, in actual surgeries the experimental setting cannot be standardized and therefore, lacks reproducibility. This brings up the potential of virtual reality (VR) simulation in technical skill assessment [72]. VR enables simulation and assessment of complex tasks, as faced in actual surgery, without exposing patients to any harm. Furthermore, the management of rare but far-reaching intraoperative adverse events like hemorrhage or vascular injury can be trained to proficiency in VR simulation.

The comparison of studies is impaired by the different scales and scores used to measure technical skill. Some studies use ordinal scales with different numbers of skill levels (good vs. bad, novice vs. intermediate vs. expert). Dichotomous classification of technical skill in good or bad performance seems obvious, however, remains highly subjective. Skill levels distinguishing novice, intermediate, and expert surgeons are often based on quantitative measures like operative volume or years in training but fail to reflect individual technical skill levels. Other studies used different interval scales (OSATS scores, GEARS scores, or Likert scales). In contrast to expert annotated or quantitatively derived skill levels, OSATS and GEARS are scores, that have proven reliability and construct validity for direct observation or video-based assessment [70, 71]. However, for the purpose of AI model training there is no standardization of skill annotation. Which part of the task, using which ontology, and in which interval technical skill should be annotated by experts to reflect the overall skill level of study participants remains to be defined.

Most of the studies included in this systematic review have methodologic limitations. Overall, 84% of studies included in this review are at RoB. The quality assessment of the included studies revealed that only 36% of the studies discussed the findings and implications in detail. Furthermore, only four studies included in this review have a multicentric dataset. Only four of the AI models studied are validated on an independent external dataset. Therefore, it is questionable whether the AI models included in this review would generalize to other settings, tasks, and institutions. Out of 50 included studies, 35 (70%) report on accuracy. However, there is a large variation of reported performance metrics among the studies included in this systematic review. Due to the novelty of AI application in the healthcare domain and in surgery in particular, the literature lacks standards in the evaluation of AI methods and their performance. There is an urgent need for the application of guidelines to assess AI models and for studies comparing them head-to-head. Guidelines for early-stage clinical evaluation of AI [73] and clinical trials involving AI [74] have been published recently. However, the studies included in this review are all at a preclinical stage where these guidelines do not apply. A multi-stakeholder initiative recently introduced guidelines and flowcharts on the choice of AI evaluation metrics in the medical image domain [75]. For surgical video analysis this effort still needs to be taken [76].

This systematic review is limited by the lack of generalizability and methodologic limitations of the included studies. Therefore, the direct comparison of AI models and a meta-analysis summarizing the evidence of included studies is not meaningful. To overcome these limitations valid and representative datasets, the use of predefined performance metrics, and external validation in clinical implementation studies will be essential to develop robust and generalizable AI models for technical skill assessment. In conclusion, AI has great potential to automate technical skill assessment in minimally invasive surgery. AI models showed moderate to high accuracy in technical skill assessment. However, the studies included in this review lack standardization of datasets, performance metrics and external validation. Therefore, we advocate for benchmarking of AI models on valid and representative datasets using predefined performance metrics and testing in clinical implementation studies.

### Supplementary Information

Below is the link to the electronic supplementary material.

Supplementary file1 (DOCX 298 KB)

## Data Availability

All data produced in the present work are contained in the manuscript.
