# Supplementary material for: Technical skill assessment in minimally invasive surgery using artificial intelligence: a systematic review
Source: Surg Endosc. 2023 Aug 16;37(10):7412–24. doi: 10.1007/s00464-023-10335-z (PMC10520175; doi:10.1007/s00464-023-10335-z)
Supplement: Supplementary file 1 — Supplementary file1 (DOCX 298 KB) [file 464_2023_10335_MOESM1_ESM.docx]

Supplementary

Technical Skill Assessment in Minimally Invasive Surgery Using Artificial Intelligence:

A Systematic Review

Romina Pedrett, MD^1^, Pietro Mascagni, MD, PhD^2,3^, Guido Beldi, MD^1^, Nicolas Padoy, PhD^2,4^, Joël L. Lavanchy, MD^1,2,5^

1 Department of Visceral Surgery and Medicine, Inselspital, Bern University Hospital, University of Bern, Switzerland

2 IHU Strasbourg, France

3 Fondazione Policlinico Universitario A. Gemelli IRCCS, Rome, Italy

4 ICube, University of Strasbourg, CNRS, France

5 University Digestive Health Care Center Basel – Clarunis, Switzerland

Correspondence and requests for reprints to:

Joël L. Lavanchy, MD

University Digestive Health Care Center Basel – Clarunis

PO Box, 4002 Basel

Switzerland

joel.lavanchy@clarunis.ch

Table S1: PRISMA Checklist

| **Section and Topic** | **Item #** | **Checklist item** | **Location where item is reported** |
| --- | --- | --- | --- |
| **TITLE** | | |  |
| Title | 1 | Identify the report as a systematic review. | p 1 |
| **ABSTRACT** | | |  |
| Abstract | 2 | See the PRISMA 2020 for Abstracts checklist. | p 2 |
| **INTRODUCTION** | | |  |
| Rationale | 3 | Describe the rationale for the review in the context of existing knowledge. | pp 4 & 5 |
| Objectives | 4 | Provide an explicit statement of the objective(s) or question(s) the review addresses. | p 5 |
| **METHODS** | | |  |
| Eligibility criteria | 5 | Specify the inclusion and exclusion criteria for the review and how studies were grouped for the syntheses. | p 6 |
| Information sources | 6 | Specify all databases, registers, websites, organisations, reference lists and other sources searched or consulted to identify studies. Specify the date when each source was last searched or consulted. | p 6 |
| Search strategy | 7 | Present the full search strategies for all databases, registers and websites, including any filters and limits used. | Supplementary |
| Selection process | 8 | Specify the methods used to decide whether a study met the inclusion criteria of the review, including how many reviewers screened each record and each report retrieved, whether they worked independently, and if applicable, details of automation tools used in the process. | pp 6 & 7 |
| Data collection process | 9 | Specify the methods used to collect data from reports, including how many reviewers collected data from each report, whether they worked independently, any processes for obtaining or confirming data from study investigators, and if applicable, details of automation tools used in the process. | p 7 |
| Data items | 10a | List and define all outcomes for which data were sought. Specify whether all results that were compatible with each outcome domain in each study were sought (e.g. for all measures, time points, analyses), and if not, the methods used to decide which results to collect. | p 7 |
|  | 10b | List and define all other variables for which data were sought (e.g. participant and intervention characteristics, funding sources). Describe any assumptions made about any missing or unclear information. | p 7 |
| Study risk of bias assessment | 11 | Specify the methods used to assess risk of bias in the included studies, including details of the tool(s) used, how many reviewers assessed each study and whether they worked independently, and if applicable, details of automation tools used in the process. | p 7 |
| Effect measures | 12 | Specify for each outcome the effect measure(s) (e.g. risk ratio, mean difference) used in the synthesis or presentation of results. | p 7 |
| Synthesis methods | 13a | Describe the processes used to decide which studies were eligible for each synthesis (e.g. tabulating the study intervention characteristics and comparing against the planned groups for each synthesis (item #5)). | p 7 |
|  | 13b | Describe any methods required to prepare the data for presentation or synthesis, such as handling of missing summary statistics, or data conversions. | na |
|  | 13c | Describe any methods used to tabulate or visually display results of individual studies and syntheses. | p 7 |
|  | 13d | Describe any methods used to synthesize results and provide a rationale for the choice(s). If meta-analysis was performed, describe the model(s), method(s) to identify the presence and extent of statistical heterogeneity, and software package(s) used. | p 7 |
|  | 13e | Describe any methods used to explore possible causes of heterogeneity among study results (e.g. subgroup analysis, meta-regression). | na |
|  | 13f | Describe any sensitivity analyses conducted to assess robustness of the synthesized results. | na |
| Reporting bias assessment | 14 | Describe any methods used to assess risk of bias due to missing results in a synthesis (arising from reporting biases). | p 8 |
| Certainty assessment | 15 | Describe any methods used to assess certainty (or confidence) in the body of evidence for an outcome. | p 8 |
| **RESULTS** | | |  |
| Study selection | 16a | Describe the results of the search and selection process, from the number of records identified in the search to the number of studies included in the review, ideally using a flow diagram. | p 9 |
|  | 16b | Cite studies that might appear to meet the inclusion criteria, but which were excluded, and explain why they were excluded. | na |
| Study characteristics | 17 | Cite each included study and present its characteristics. | pp 9-13 |
| Risk of bias in studies | 18 | Present assessments of risk of bias for each included study. | Figure 4 &  Supplementary |
| Results of individual studies | 19 | For all outcomes, present, for each study: (a) summary statistics for each group (where appropriate) and (b) an effect estimate and its precision (e.g. confidence/credible interval), ideally using structured tables or plots. | pp 9-13 |
| Results of syntheses | 20a | For each synthesis, briefly summarise the characteristics and risk of bias among contributing studies. | na |
|  | 20b | Present results of all statistical syntheses conducted. If meta-analysis was done, present for each the summary estimate and its precision (e.g. confidence/credible interval) and measures of statistical heterogeneity. If comparing groups, describe the direction of the effect. | pp 9-13 |
|  | 20c | Present results of all investigations of possible causes of heterogeneity among study results. | na |
|  | 20d | Present results of all sensitivity analyses conducted to assess the robustness of the synthesized results. | na |
| Reporting biases | 21 | Present assessments of risk of bias due to missing results (arising from reporting biases) for each synthesis assessed. | na |
| Certainty of evidence | 22 | Present assessments of certainty (or confidence) in the body of evidence for each outcome assessed. | na |
| **DISCUSSION** | | |  |
| Discussion | 23a | Provide a general interpretation of the results in the context of other evidence. | p 15 |
|  | 23b | Discuss any limitations of the evidence included in the review. | pp 15 & 16 |
|  | 23c | Discuss any limitations of the review processes used. | p 17 |
|  | 23d | Discuss implications of the results for practice, policy, and future research. | pp 16 & 17 |
| **OTHER INFORMATION** | | |  |
| Registration and protocol | 24a | Provide registration information for the review, including register name and registration number, or state that the review was not registered. | p 6 |
|  | 24b | Indicate where the review protocol can be accessed, or state that a protocol was not prepared. | p 6 |
|  | 24c | Describe and explain any amendments to information provided at registration or in the protocol. | na |
| Support | 25 | Describe sources of financial or non-financial support for the review, and the role of the funders or sponsors in the review. | p 18 |
| Competing interests | 26 | Declare any competing interests of review authors. | p 18 |
| Availability of data, code and other materials | 27 | Report which of the following are publicly available and where they can be found: template data collection forms; data extracted from included studies; data used for all analyses; analytic code; any other materials used in the review. | Supplementary |

*From:*  Page MJ, McKenzie JE, Bossuyt PM, Boutron I, Hoffmann TC, Mulrow CD, et al. The PRISMA 2020 statement: an updated guideline for reporting systematic reviews. BMJ 2021;372:n71. doi: 10.1136/bmj.n71

For more information, visit: <http://www.prisma-statement.org/>

Table S2: Full-text search terms of the search strategy

Definitive Literature Search Strategy conducted by Tanya Karrer, Information Specialist Medicine, Research Support Services, University Library, University of Bern, August 25^th^ 2021, re-runs February 25^th^ 2022 and May 31^st^ 2023.

**OVID MEDLINE**(R) and Epub Ahead of Print, In-Process, In-Data-Review & Other Non-Indexed Citations, Daily and Versions(R) < 1946 to August 24, 2021 > . 25.8.2021

1 exp Minimally Invasive Surgical Procedures/ 551322

2 Robotic Surgical Procedures/ 10673

3 Surgery, Computer-Assisted/ 18834

4 Specialties, Surgical/ 4298

5 Surgeons/ed [Education] 1043

6 (Laparoscop* or minimal-invasive surg* or minimally invasive surg* or celioscop* or peritoneoscop* or box-model* or box model* or remote operation* or telerobotic* or tele-robotic* or tele robotic* or robot* surg* or robot-assisted surg* or robot assisted surg* or robot-enhanced surg* or robot enhanced surg* or surgical robot* or surgery robot* or computer-assisted surg* or computer assisted surg* or surg* education).tw. 155871

7 or/1-6 627683

8 artificial intelligence/ or machine learning/ or deep learning/ or supervised machine learning/ or unsupervised machine learning/ or neural networks, computer/ or Pattern Recognition, Automated/ 89828

9 (artificial intelligence or artificial surg* or neural network* or comput* network model* or machine learning or machine-learning or deep learning* or deep-learning or natural language process* or motion tracking* or automat* pattern recognition* or comput* intelligence or machine intelligence or computer reasoning or computational reasoning or computer vision* or computational vision* or machine sensing).tw. 121663

10 8 or 9 162542

11 ((surgical or surgeon* or laparoscopic or clinical) adj6 (skill* or performance or technique* or quality or process* or model* or level*) adj6 (assess* or evaluat* or classification or rating or scor* or accuracy)).tw. 41471

12 Clinical competence/ 98831

13 Professionalism/ed [Education] 163

14 Education, Medical, Continuing/ 25232

15 Laparoscopy/ed [Education] 1794

16 Surgeons/ed [Education] 1043

17 "Task Performance and Analysis"/ 32098

18 11 or 12 or 13 or 14 or 15 or 16 or 17 188918

19 7 and 10 and 18 212

20 limit 19 to last 5 years

**Results: 142**

<https://ovidsp.ovid.com/ovidweb.cgi?T=JS&NEWS=N&PAGE=main&SHAREDSEARCHID=6mwmKdYyxRIM7S9MsMe1vGNE323wr8aTObZxUuKBd8V9KCAB4xRFCZZhYcQXls1eE>

**EMBASE VIA OVID** <1974 to 2021 August 24 >, 25.8.2021

1 exp minimally invasive surgery/ 46081

2 exp computer assisted surgery/ 24547

3 exp laparoscopic surgery/ 89074

4 exp laparoscopy/ 172635

5 surgical technology/ 2054

6 (Laparoscop* or minimal-invasive surg* or minimally invasive surg* or celioscop* or peritoneoscop* or box-model* or box model* or remote operation* or telerobotic* or tele-robotic* or tele robotic* or robot* surg* or robot-assisted surg* or robot assisted surg* or robot-enhanced surg* or robot enhanced surg* or surgical robot* or surgery robot* or computer-assisted surg* or computer assisted surg* or surg* education).ab,kw,ti. 255614

7 1 or 2 or 3 or 4 or 5 or 6 323807

8 artificial intelligence/ 33832

9 deep learning/ 17462

10 supervised machine learning/ 2224

11 unsupervised machine learning/ 1173

12 machine learning/ or exp artificial neural network/ or automated pattern recognition/ or network learning/ 112821

13 (artificial intelligence or artificial surg* or neural network* or comput* network model* or machine learning or machine-learning or deep learning* or deep-learning or natural language process* or motion tracking* or automat* pattern recognition* or comput* intelligence or machine intelligence or computer reasoning or computational reasoning or computer vision* or computational vision* or machine sensing).ti,ab,kw. 156778

14 8 or 9 or 10 or 11 or 12 or 13 199420

15 clinical competence/ 63852

16 professionalism/ 9841

17 continuing education/ 31781

18 exp surgical training/ 22826

19 task performance/ 147487

20 ((surgical or surgeon* or laparoscopic or clinical) adj6 (skill* or performance or technique* or quality or process* or model* or level*) adj6 (assess* or evaluat* or classification or rating or scor* or accuracy)).ti,ab,kw. 60521

21 15 or 16 or 17 or 18 or 19 or 20 326609

22 7 and 14 and 21 262

23 limit 22 to last 5 years

**Results: 189**

[https://ovidsp.ovid.com/ovidweb.cgi?T=JS&NEWS=N&PAGE=main&SHAREDSEARCHID=2va9FFfK1QL 2YjNsjNzGNRax2EXAD4RKfK1aLDyojM2ST9e8mUAeL3Xvnz7xD4tOh](https://ovidsp.ovid.com/ovidweb.cgi?T=JS&NEWS=N&PAGE=main&SHAREDSEARCHID=2va9FFfK1QL%202YjNsjNzGNRax2EXAD4RKfK1aLDyojM2ST9e8mUAeL3Xvnz7xD4tOh)

**WEB OF SCIENCE**, 25.8.2021

((TS=(Laparoscop* or minimal-invasive surg* or minimally invasive surg* or celioscop* or peritoneoscop* or box-model* or box model* or remote operation* or telerobotic* or telerobotic* or tele robotic* or robot* surg* or robot-assisted surg* or robot assisted surg* or robotenhanced surg* or robot enhanced surg* or surgical robot* or surgery robot* or computerassisted surg* or computer assisted surg* or surg* education or surg* training or surg* technolog*)) AND TS=(artificial intelligence or artificial surg* or neural network* or comput* network model* or machine learning or machine-learning or deep learning* or deep-learning* or natural language process* or motion tracking* or automat* pattern recognition* or comput* intelligence or machine intelligence or computer reasoning or computational reasoning or computer vision* or computational vision* or machine sensing)) AND TS=((surgical OR surgeon* OR laparoscopic OR clinical) NEAR/6 (skill* OR performance OR technique* OR quality OR process* OR model* OR level*) NEAR/6 (assess* OR evaluat* OR classification OR rating* OR scoring* OR accuracy)) AND DOP=(2016-01-01/2021-12-31)

**Results: 265**

<https://www.webofscience.com/wos/woscc/summary/97217178-1812-43fe-a337-6ffa3e14978e-057db63a/relevance/1>

**IEEE XPLORE**, 25.8.2021

In all Metadata (Includes the abstract, index terms, and bibliographic citation data (such as document title, publication title, author, etc.) ((((("All Metadata":Laparoscop* OR "All Metadata":"minimally invasive surgery" OR "All Metadata":"minimal-invasive surgery" OR "All Metadata":robot* surg* OR "All Metadata":"remote operation*" OR "All Metadata":"computer assisted surgery" OR "All Metadata":"box-model")))) AND ((("All Metadata":skill* OR "All Metadata":performance OR "All Metadata":technique* OR "All Metadata":quality OR "All Metadata":level OR "All Metadata":assessment OR "All Metadata":evaluation OR "All Metadata":classification OR "All Metadata":rating OR "All Metadata":scoring OR "All Metadata":accuracy)))) AND ((("All Metadata":"artificial intelligence" OR "All Metadata":machine learning OR "All Metadata":"deep learning" OR "All Metadata":"deeplearning" OR "All Metadata":"natural language processing" OR "All Metadata":"computer vision" OR "All Metadata":"machine sensing" OR "All Metadata":"neural network" OR "All Metadata":"neural networks")))

Filter: Conferences, Journals, Early Access Articles, Standards, Years 2016-2021

**Results: 583**

<https://ieeexplore.ieee.org/search/searchresult.jsp?contentType=all&refinements=ContentType%3AConferences&refinements=ContentType%3AJournals&refinements=ContentType%3AEarly+Access+Articles&refinements=ContentType%3AStandards&sortType=&ranges=2016_2021_PublicationYear&matchBoolean=true&searchField=Search_All&queryText=((((((Search_All:Laparoscop*+OR+Search_All:%22minimally+invasive+surgery%22+OR+Search_All:%22minimal-invasive+surgery%22+OR+Search_All:robot*+surg*+OR+Search_All:%22remote+operation*%22+OR+Search_All:%22computer+assisted+surgery%22+OR+Search_All:%22box-model%22))))+AND+(((Search_All:skill*+OR+Search_All:performance+OR+Search_All:technique*+OR+Search_All:quality+OR+Search_All:level+OR+Search_All:assessment+OR+Search_All:evaluation+OR+Search_All:classification+OR+Search_All:rating+OR+Search_All:scoring+OR+Search_All:accuracy))))+AND+(((Search_All:%22artificial+intelligence%22+OR+Search_All:machine+learning+OR+Search_All:%22deep+learning%22+OR+Search_All:%22deep-learning%22+OR+Search_All:%22natural+language+processing%22+OR+Search_All:%22computer+vision%22+OR+Search_All:%22machine+sensing%22+OR+Search_All:%22neural+network%22+OR+Search_All:%22neural+networks%22))))&history=no>

Table S3: Data extracted from the 50 studies included in this systematic review

| Title | Authors | Year | Country | Journal | Population | Setting | Tasks | Input Data | AI Model | Assessment | Results |  |  |  |  |  |  |  |  |
| --- | --- | --- | --- | --- | --- | --- | --- | --- | --- | --- | --- | --- | --- | --- | --- | --- | --- | --- | --- |
| Development of a Laparoscopic Box Trainer Based on Open Source Hardware and Artificial Intelligence for Objective Assessment of Surgical Psychomotor Skills[1] | Gustavo A. Alonso-Silverio, Fernando Pérez-Escamirosa, Raúl Bruno-Sanchez, José L. Ortiz-Simon, Roberto Muñoz-Guerrero, Arturo Minor-Martinez, Antonio Alarcón-Paredes | 2018 | Mexico | Surgical Innovation | 20 volunteers | LS | PC | VR | ANN | binary (experienced, non-experienced) | Validation scheme | Accuracy | Sensitivity | Specificity | AUC |  |  |  |  |
|  |  |  |  |  |  |  |  |  |  |  | Holdout (67/33) | 93.94% | 0.78 | 1 | 0.97 |  |  |  |  |
|  |  |  |  |  |  |  |  |  |  |  | 10-fold cross-validation | 91% | 0.78 | 0.96 | 0.93 |  |  |  |  |
|  |  |  |  |  |  |  |  |  |  |  | Leave one out | 88% | 0.74 | 0.93 | 0.91 |  |  |  |  |
| Keep Your Eye on the Best: Contrastive Regression Transformer for Skill Assessment in Robotic Surgery[2] | Dimitrios Anastasiou, Yueming Jin, Danail Stoyanov, Evangelos Mazomenos | 2023 | U.K. | IEEE Robotics and Automation Letters | 8 participants | RS | SU, NP, KT | VR | DL (ResNet-18) | GRS Score | Spearman's 0.65–0.89 | Normalized MAE 5.8-13.4% |  |  |  |  |  |  |  |
|  |  |  |  |  |  |  |  |  |  |  |  | KT |  |  | NP |  |  |  |  |
|  |  |  |  |  |  |  |  |  |  |  |  | LOSO | LOUO | 4-Fold | LOSO | LOUO | 4-Fold |  |  |
|  |  |  |  |  |  |  |  |  |  |  | Spearman’s Correlation Coefficient (SCC) | 0.89 | 0.69 | 0.87 | 0.71 | 0.71 | 0.81 |  |  |
|  |  |  |  |  |  |  |  |  |  |  | Mean Absolute Error (MAE) | 1.75 | 1.39 | 2.1 | 3.15 | 3.17 | 3.21 |  |  |
|  |  |  |  |  |  |  |  |  |  |  |  | SU |  |  | Across Tasks |  |  |  |  |
|  |  |  |  |  |  |  |  |  |  |  |  | LOSO | LOUO | 4-Fold | LOSO | LOUO | 4-Fold |  |  |
|  |  |  |  |  |  |  |  |  |  |  | Spearman’s Correlation Coefficient (SCC) | 0.86 | 0.65 | 0.69 | 0.83 | 0.68 | 0.81 |  |  |
|  |  |  |  |  |  |  |  |  |  |  | Mean Absolute Error (MAE) | 2.74 | 2.58 | 2.99 | 2.55 | 2.38 | 2.77 |  |  |
| Towards near real-time assessment of surgical skills: A comparison of feature extraction techniques[3] | Nguyen Xuan Anh, Ramesh Mark Nataraja, Sunita Chauhan | 2020 | Australia | Computer Methods and Programs in Biomedicine | 8 participants | RS | SU, NP, KT | KD (dV) | DL, CNN | N, I, E | Method | 3s |  | 4s |  | 5s |  | 6s |  |
|  |  |  |  |  |  |  |  |  |  |  |  | 1s | 2s | 1s | 2s | 1s | 2s | 1s | 2s |
|  |  |  |  |  |  |  |  |  |  |  | Suturing task: Accuracy (effect of window size) |  |  |  |  |  |  |  |  |
|  |  |  |  |  |  |  |  |  |  |  | CNN | 96.84 | 96.84 | 96.9 | 96.87 | 96.92 | 96.82 | 97.04 | 96.92 |
|  |  |  |  |  |  |  |  |  |  |  | LSTM | 95.09 | 94.57 | 96.39 | 95.67 | 96.45 | 95.8 | 96.48 | 96.45 |
|  |  |  |  |  |  |  |  |  |  |  | CNN-LSTM | 96.39 | 96.3 | 96.44 | 96.19 | 96.64 | 96.05 | 96.69 | 96.16 |
|  |  |  |  |  |  |  |  |  |  |  | Auto-encoder | 83.46 | 79.81 | 80.7 | 79.94 | 81 | 80.69 | 81.04 | 80.96 |
|  |  |  |  |  |  |  |  |  |  |  | Needle passing |  |  |  |  |  |  |  |  |
|  |  |  |  |  |  |  |  |  |  |  | CNN | 95.36 | 94.96 | 95.65 | 95.12 | 95.83 | 95.46 | 96.44 | 95.88 |
|  |  |  |  |  |  |  |  |  |  |  | LSTM | 91.52 | 90.67 | 93.63 | 92.19 | 93.83 | 92.94 | 94.06 | 93.05 |
|  |  |  |  |  |  |  |  |  |  |  | CNN-LSTM | 93.45 | 92.26 | 93.05 | 92.73 | 93.44 | 93.16 | 94.2 | 94.11 |
|  |  |  |  |  |  |  |  |  |  |  | Auto-encoder | 82.25 | 80.33 | 80.36 | 80.18 | 80.13 | 79.98 | 81.5 | 80.25 |
|  |  |  |  |  |  |  |  |  |  |  | Knot tying |  |  |  |  |  |  |  |  |
|  |  |  |  |  |  |  |  |  |  |  | CNN | 92.75 | 92.16 | 93.02 | 92.94 | 93.13 | 93.51 | 93.56 | 93.49 |
|  |  |  |  |  |  |  |  |  |  |  | LSTM | 89.57 | 89.38 | 91.31 | 89.88 | 91.41 | 89.94 | 91.56 | 90.36 |
|  |  |  |  |  |  |  |  |  |  |  | CNN-LSTM | 90.98 | 90.24 | 91.74 | 90.25 | 91.95 | 90.53 | 93.01 | 91.1 |
|  |  |  |  |  |  |  |  |  |  |  | Auto-encoder | 80.63 | 76.02 | 77.75 | 75.95 | 77.6 | 75.15 | 77.69 | 75.54 |
| A computer vision technique for automated assessment of surgical performance using surgeons' console-feed videos[4] | Amir Baghdadi, Ahmed A. Hussein, Youssef Ahmed, Lora A. Cavuoto, Khurshid A. Guru | 2018 | USA | International Journal of Computer Assisted Radiology and Surgery | unknown | Lap | Pelvic lymph node dissection | VR | CV, ML (Logistic regression) | PLACE (pelvic lymphadenectomy appropriateness and completion evaluation) | Accuracy | 0.833 |  |  |  |  |  |  |  |
| A neural network architecture for automatic and objective surgical skill assessment[5] | Malik Benmansour, Wahida Handouzi, Abed Malti | 2018 | Algeria | PROCEEDINGS 2018 3RD INTERNATIONAL CONFERENCE ON ELECTRICAL SCIENCES AND TECHNOLOGIES IN MAGHREB (CISTEM) | 6 participants  (2 intermediates,  4 novices) | RS | SU, NP, KT | KD (dV) | DNN | Custom score | not available |  |  |  |  |  |  |  |  |
| Deep neural network architecture for automated soft surgical skills evaluation using objective structured assessment of technical skills criteria[6] | Malik Benmansour, Abed Malti, Pierre Jannin | 2023 | Algeria | International Journal of Computer Assisted Radiology and Surgery | 8 participants | RS | SU, NP, KT | KD (dV) | DL (CNN+ BiLSTM) | OSATS score |  | KT | NP | SU |  |  |  |  |  |
|  |  |  |  |  |  |  |  |  |  |  | LOSO |  |  |  |  |  |  |  |  |
|  |  |  |  |  |  |  |  |  |  |  | Spearman's coefficient | 0.82 | 0.6 | 0.65 |  |  |  |  |  |
|  |  |  |  |  |  |  |  |  |  |  | Mean Squared Error | 0.25 | 0.47 | 0.44 |  |  |  |  |  |
| Using Contact Forces and Robot Arm Accelerations to Automatically Rate Surgeon Skill at Peg Transfer[7] | Jeremy D. Brown, Conor E. O’Brien, Sarah C. Leung, Kristoffel R. Dumon, David I. Lee, and Katherine J. Kuchenbecker | 2017 | USA | IEEE transactions on bio-medical engineering | 38 participants  (3 trials each) | RS | PT | KD (dV) | ML | GEARS scores (1-5: exact rating) | Accuracy | GEARS domain | Regression learner | Classification learner |  |  |  |  |  |
|  |  |  |  |  |  |  |  |  |  |  |  | Depth perception | 63.3 +/- 9.5% | 71.7 +/- 9.5% |  |  |  |  |  |
|  |  |  |  |  |  |  |  |  |  |  |  | Bimanual dexterity | 66.7 +/- 11.8% | 53.3 +/- 16.2% |  |  |  |  |  |
|  |  |  |  |  |  |  |  |  |  |  |  | Efficiency | 73.3 +/-16.0% | 58.3 +/- 8.3% |  |  |  |  |  |
|  |  |  |  |  |  |  |  |  |  |  |  | Force sensitivity | 63.3 +/- 9.5% | 51.7 +/- 10.9% |  |  |  |  |  |
|  |  |  |  |  |  |  |  |  |  |  |  | Robotic control | 71.7 +/- 12.6% | 75.0 +/- 15.6% |  |  |  |  |  |
|  |  |  |  |  |  |  |  |  |  |  | Precision |  |  |  |  |  |  |  |  |
|  |  |  |  |  |  |  |  |  |  |  | Regression | Depth perception | 0.47 +/- 0.37 |  |  |  |  |  |  |
|  |  |  |  |  |  |  |  |  |  |  |  | Bimanual dexterity | 0.53 +/- 0.38 |  |  |  |  |  |  |
|  |  |  |  |  |  |  |  |  |  |  |  | Efficiency | 0.64 +/- 0.36 |  |  |  |  |  |  |
|  |  |  |  |  |  |  |  |  |  |  |  | Force sensitivity | 0.43 +/- 0.39 |  |  |  |  |  |  |
|  |  |  |  |  |  |  |  |  |  |  |  | Robotic control | 0.47 +/- 0.40 |  |  |  |  |  |  |
|  |  |  |  |  |  |  |  |  |  |  | Classification | Depth perception | 0.40 +/- 0.40 |  |  |  |  |  |  |
|  |  |  |  |  |  |  |  |  |  |  |  | Bimanual dexterity | 0.28 +/- 0.30 |  |  |  |  |  |  |
|  |  |  |  |  |  |  |  |  |  |  |  | Efficiency | 0.50 +/- 0.38 |  |  |  |  |  |  |
|  |  |  |  |  |  |  |  |  |  |  |  | Force sensitivity | 0.26 +/- 0.28 |  |  |  |  |  |  |
|  |  |  |  |  |  |  |  |  |  |  |  | Robotic control | 0.44 +/- 0.43 |  |  |  |  |  |  |
|  |  |  |  |  |  |  |  |  |  |  | Recall |  |  |  |  |  |  |  |  |
|  |  |  |  |  |  |  |  |  |  |  | Regression | Depth perception | 0.56 +/- 0.38 |  |  |  |  |  |  |
|  |  |  |  |  |  |  |  |  |  |  |  | Bimanual dexterity | 0.55 +/- 0.38 |  |  |  |  |  |  |
|  |  |  |  |  |  |  |  |  |  |  |  | Efficiency | 0.65 +/- 0.36 |  |  |  |  |  |  |
|  |  |  |  |  |  |  |  |  |  |  |  | Force sensitivity | 0.45 +/- 0.41 |  |  |  |  |  |  |
|  |  |  |  |  |  |  |  |  |  |  |  | Robotic control | 0.55 +/- 0.44 |  |  |  |  |  |  |
|  |  |  |  |  |  |  |  |  |  |  | Classification | Depth perception | 0.48 +/- 0.47 |  |  |  |  |  |  |
|  |  |  |  |  |  |  |  |  |  |  |  | Bimanual dexterity | 0.38 +/- 0.41 |  |  |  |  |  |  |
|  |  |  |  |  |  |  |  |  |  |  |  | Efficiency | 0.54 +/- 0.33 |  |  |  |  |  |  |
|  |  |  |  |  |  |  |  |  |  |  |  | Force sensitivity | 0.37 +/- 0.40 |  |  |  |  |  |  |
|  |  |  |  |  |  |  |  |  |  |  |  | Robotic control | 0.51 +/- 0.47 |  |  |  |  |  |  |
| Towards Optimizing Convolutional Neural Networks for Robotic Surgery Skill Evaluation[8] | Dayvid Castro, Danilo Pereira, Cleber Zanchettin, David Macêdo, Byron L. D. Bezerra | 2019 | Brazil | International Joint Conference on Neural Networks | 8 participants | RS | SU, NP, KT | KD (dV) | CNN | N, I, E |  | Mikro-accuracy | Makro-accuracy |  |  |  |  |  |  |
|  |  |  |  |  |  |  |  |  |  |  | Suturing task | 98.37 | 98.65 |  |  |  |  |  |  |
|  |  |  |  |  |  |  |  |  |  |  | Needle-passing | 98.89 | 98.98 |  |  |  |  |  |  |
|  |  |  |  |  |  |  |  |  |  |  | Knot-tying | 98.9 | 98.68 |  |  |  |  |  |  |
| Automated robot‐assisted surgical skill evaluation: Predictive analytics approach[9] | Mahtab J. Fard, Sattar Ameri, R. Darin Ellis, Ratna B. Chinnam, Abhilash K. Pandya, Michael D. Klein | 2017 | USA | The International Journal of Medical Robotics and Computer Assisted Surgery | 8 participants | RS | SU, KT | KD (dV) | ML | binary (N, E) | Accuracy | LOSO (LR best) | LOUO (SVM best) |  |  |  |  |  |  |
|  |  |  |  |  |  |  |  |  |  |  | Suturing task | 89.9 | 79.8 |  |  |  |  |  |  |
|  |  |  |  |  |  |  |  |  |  |  | Knot-tying | 82.3 | 77.9 |  |  |  |  |  |  |
| Surgical Skill Assessment System Using Fuzzy Logic in a Multi-Class Detection of Laparoscopic Box-Trainer Instruments[10] | Fatemeh Rashidi Fathabadi, Janos L. Grantner, Saad A Shebrain, Ikhlas Abdel-Qader | 2021 | USA | IEEE International Conference on Systems, Man, and Cybernetics (SMC) | unknown | LS | PC | VR | DL | Levels A (excellent)-E (very bad) | not available |  |  |  |  |  |  |  |  |
| Accurate and interpretable evaluation of surgical skills from kinematic data using fully convolutional neural networks[11] | Hassan Ismail Fawaz, Germain Forestier, Jonathan Weber, Lhassane Idoumghar, Pierre-Alain Muller | 2019 | France | International Journal of Computer Assisted Radiology and Surgery | 8 participants | RS | SU, NP, KT | KD (dV) | CNN | N, I, E |  | Mikro-accuracy | Makro-accuracy | Spearman's Coefficient |  |  |  |  |  |
|  |  |  |  |  |  |  |  |  |  |  | Suturing task | 100 | 100 | 0.6 |  |  |  |  |  |
|  |  |  |  |  |  |  |  |  |  |  | Needle-passing | 100 | 100 | 0.57 |  |  |  |  |  |
|  |  |  |  |  |  |  |  |  |  |  | Knot-tying | 92.1 | 93.2 | 0.65 |  |  |  |  |  |
| Surgical motion analysis using discriminative interpretable patterns[12] | Germain Forestier, François Petitjean, Pavel Senin, Fabien Despinoy, Arnaud Huaulmé, Hassan Ismail Fawaz, Jonathan Weber, Lhassane Idoumghar, Pierre-Alain Muller, Pierre Jannin | 2018 | France | Artificial Intelligence In Medicine | 8 participants | RS | SU, NP, KT | KD (dV) | ML | N, I, E |  | Mikro-accuracy/Precision (LOSO) | Makro-accuracy/Precision (LOSO) |  |  |  |  |  |  |
|  |  |  |  |  |  |  |  |  |  |  | Suturing task | 89.74 | 86.67 |  |  |  |  |  |  |
|  |  |  |  |  |  |  |  |  |  |  | Needle-passing | 96.3 | 95.83 |  |  |  |  |  |  |
|  |  |  |  |  |  |  |  |  |  |  | Knot-tying | 61.11 | 53.33 |  |  |  |  |  |  |
| Predicting surgical skill from the first N seconds of a task: value over task time using the isogony principle[13] | Anna French, Thomas S. Lendvay, Robert M. Sweet, Timothy M. Kowalewski | 2017 | USA | International Journal of Computer Assisted Radiology and Surgery | 98 surgeons | LS | PT, SU, PC | VR | ML | N, E / N, I, E | Accuracy (2-class classification) | Peg transfer | Cutting | Suturing | Best % accuracy mean/median | Min time to 90% of best accuracy |  |  |  |
|  |  |  |  |  |  |  |  |  |  |  | DA | 80.1 (0.13) | 86.1 (0.11) | 83.0 (0.2) | 83.7/85.7 | 2 s |  |  |  |
|  |  |  |  |  |  |  |  |  |  |  | QDA | 87.6 (0.12) | 86.9 (0.11) | 66.0 (0.24) | 82.5/82.6 | 4 s |  |  |  |
|  |  |  |  |  |  |  |  |  |  |  | SVM | 85.2 (0.12) | 90.2 (0.10) | 82.6 (0.20) | 87.2/88.7 | 8 s |  |  |  |
|  |  |  |  |  |  |  |  |  |  |  | LR | 84.6 (0.12) | 90.2 (0.10) | 81.5 (0.21) | 86.6/86.4 | 7 s |  |  |  |
|  |  |  |  |  |  |  |  |  |  |  | Accuracy (3-class classification) | Peg transfer | Cutting | Suturing | Best % accuracy mean/median | Min time to 90% of best accuracy |  |  |  |
|  |  |  |  |  |  |  |  |  |  |  | LDA = linear discriminant analysis | 57.3 (0.13) | 63.0 (0.14) | 66.0 (0.16) | 61.6/61.7 | 2 s |  |  |  |
|  |  |  |  |  |  |  |  |  |  |  | QDA = quadratic discriminant analysis | 66.6 (0.12) | 59.2 (0.14) | 44.3 (0.16) | 58.9/58.6 | 4 s |  |  |  |
|  |  |  |  |  |  |  |  |  |  |  | SVM | 62.2 (0.13) | 67.0 (0.13) | 65.5 (0.14) | 65.1/65.5 | 3 s |  |  |  |
|  |  |  |  |  |  |  |  |  |  |  | LR = logistic regression | 60.8 (0.12) | 68.3 (0.13) | 65.7 (0.15) | 65.1/64.3 | 3 s |  |  |  |
| Video-based surgical skill assessment using 3D convolutional neural networks[14] | Isabel Funke, Sören Torge Mees, Jürgen Weitz, Sefanie Speidel | 2019 | Germany | International Journal of Computer Assisted Radiology and Surgery | 8 participants | RS | SU, NP, KT | VR | DL, CNN | N, I, E | Knot tying | Accuracy | Avg. Recall | Avg. F1 |  |  |  |  |  |
|  |  |  |  |  |  |  |  |  |  |  | 3D ConvNet (RGB) | 95.8 +/- 1.6 | 95.6 +/- 1.2 | 95.9 +/- 1.5 |  |  |  |  |  |
|  |  |  |  |  |  |  |  |  |  |  | 3D ConvNet (OF) | 95.1 +/- 2.7 | 94.2 +/- 3.2 | 95.0 +/- 2.9 |  |  |  |  |  |
|  |  |  |  |  |  |  |  |  |  |  | Suturing | Accuracy | Avg. Recall | Avg. F1 |  |  |  |  |  |
|  |  |  |  |  |  |  |  |  |  |  | 3D ConvNet (RGB) | 100 +/- 0 | 100 +/- 0 | 100 +/- 0 |  |  |  |  |  |
|  |  |  |  |  |  |  |  |  |  |  | 3D ConvNet (OF) | 100 +/- 0 | 100 +/- 0 | 100 +/- 0 |  |  |  |  |  |
|  |  |  |  |  |  |  |  |  |  |  | Needle passing | Accuracy | Avg. Recall | Avg. F1 |  |  |  |  |  |
|  |  |  |  |  |  |  |  |  |  |  | 3D ConvNet (RGB) | 96.4 +/- 0 | 96.3 +/- 0 | 96.6 +/- 0 |  |  |  |  |  |
|  |  |  |  |  |  |  |  |  |  |  | 3D ConvNet (OF) | 100 +/- 0 | 100 +/- 0 | 100 +/- 0 |  |  |  |  |  |
| Functional Brain Imaging Reliably Predicts Bimanual Motor Skill Performance in a Standardized Surgical Task[15] | Yuanyuan Gao, Pingkun Yan, Uwe Kruger, Lora Cavuoto, Steven Schwaitzberg, Suvranu De, Xavier Intes | 2020 | USA | IEEE transactions on bio-medical engineering | 13 medical students | LS | PC | fNIRS data (functional near-infrared spectroscopy) | DL | FLS (Fundamentals of Laparoscopic Surgery) Score: Pass/fail |  | Accuracy | Sensitivity | Specificity | Computation time (ms) |  |  |  |  |
|  |  |  |  |  |  |  |  |  |  |  | Brain-NET | 0.91 | 0.95 | 0.67 | 38 |  |  |  |  |
|  |  |  |  |  |  |  |  |  |  |  | KPLS | 0.9 | 0.95 | 0.57 | 115 |  |  |  |  |
|  |  |  |  |  |  |  |  |  |  |  | SVR | 0.91 | 0.97 | 0.56 | 59 |  |  |  |  |
|  |  |  |  |  |  |  |  |  |  |  | RF | 0.9 | 0.98 | 0.33 | 109 |  |  |  |  |
| Affordable, web-based surgical skill training and evaluation tool[16] | Gazi Islam, Kanav Kahol, Baoxin Li, Marshall Smith, Vimla L. Patel | 2016 | USA | Journal of Biomedical Informatics | 52 subjects (medical students and surgical residents) | LS | PT, SU, PC | VR | CV, NN | Custom score | not available |  |  |  |  |  |  |  |  |
| Tool Detection and Operative Skill Assessment in Surgical Videos Using Region-Based Convolutional Neural Networks[17] | Amy Jin, Serena Yeung, Jeffrey Jopling, Jonathan Krause, Dan Azagury, Amy Jin, Serena Yeung, Jeffrey Jopling, Jonathan Krause, Dan Azagury, Arnold Milstein, Li Fei-Fei | 2018 | USA | [2018 IEEE Winter Conference on Applications of Computer Vision (WACV)](https://ieeexplore.ieee.org/xpl/conhome/8345804/proceeding) | unknown | Lap | Laparoscopic cholecystectomy | VR | CNN | Qualitative description | not applicable |  |  |  |  |  |  |  |  |
| Machine Learning based Classification of Local Robotic Surgical Skills in a Training Tasks Set[18] | L. Juarez-Villalobos, N. Hevia-Montiel, J. Perez-Gonzalez | 2021 | Mexico | 2021 43rd Annual International Conference of the IEEE Engineering in Medicine & Biology Society (EMBC) | 8 participants | RS | SU, NP, KT | KD (dV) | ML | binary (N, E) | Task | 10-fold cross validation |  |  | Final Test |  |  |  |  |
|  |  |  |  |  |  |  |  |  |  |  | Knot tying | Accuracy | AUC-ROC | F1 Score | Accuracy | AUC-ROC | F1 Score |  |  |
|  |  |  |  |  |  |  |  |  |  |  | KNN | 1.0 +- 0.0 | 1.0 +- 0.0 | 1.0 +- 0.0 | 1 | 1 | 1 |  |  |
|  |  |  |  |  |  |  |  |  |  |  | RF (random forest) | 1.0 +- 0.0 | 1.0 +- 0.0 | 1.0 +- 0.0 | 1 | 1 | 1 |  |  |
|  |  |  |  |  |  |  |  |  |  |  | SVM | 1.0 +- 0.0 | 1.0 +- 0.0 | 1.0 +- 0.0 | 1 | 1 | 1 |  |  |
|  |  |  |  |  |  |  |  |  |  |  | Needle-Passing |  |  |  |  |  |  |  |  |
|  |  |  |  |  |  |  |  |  |  |  | KNN | 1.0 +- 0.0 | 1.0 +- 0.0 | 1.0 +- 0.0 | 0.83 | 0.83 | 0.83 |  |  |
|  |  |  |  |  |  |  |  |  |  |  | RF (random forest) | 0.95 +- 0.16 | 0.93+-0.19 | 0.86+-0.38 | 1 | 1 | 1 |  |  |
|  |  |  |  |  |  |  |  |  |  |  | SVM | 1.0 +- 0.0 | 1.0 +- 0.0 | 1.0 +- 0.0 | 1 | 1 | 1 |  |  |
|  |  |  |  |  |  |  |  |  |  |  | Suturing |  |  |  |  |  |  |  |  |
|  |  |  |  |  |  |  |  |  |  |  | KNN | 1.0 +- 0.0 | 1.0 +- 0.0 | 1.0 +- 0.0 | 1 | 1 | 1 |  |  |
|  |  |  |  |  |  |  |  |  |  |  | RF (random forest) | 0.97+- 0.11 | 0.97+- 0.09 | 0.96+- 0.12 | 1 | 1 | 1 |  |  |
|  |  |  |  |  |  |  |  |  |  |  | SVM | 1.0 +- 0.0 | 1.0 +- 0.0 | 1.0 +- 0.0 | 1 | 1 | 1 |  |  |
| High density optical neuroimaging predicts surgeons’s subjective experience and skill levels[19] | Hasan Onur Keles, Canberk Cengiz, Irem Demiral, Mehmet Mahir Ozmen, Ahmet Omurtag | 2021 | Turkey | PLoS ONE | 16 surgeons, 17 medical students | LS | PT, threading | NIRS (functional neuroimaging data) | ML | binary (student vs. attending) | ~90% accuracy (precise results not available) |  |  |  |  |  |  |  |  |
| Bidirectional long short-term memory for surgical skill classification of temporally segmented tasks[20] | Jason D. Kelly, Ashley Petersen, Thomas S. Lendvay, Timothy M. Kowalewski | 2020 | USA | International Journal of Computer Assisted Radiology and Surgery | unknown | LS | 110 PT, 110 PC, 115 SU, 119 clipping | VR | CNN | binary (N, E) |  | Accuracy | Novice-specific accuracy | Expert-specific accuracy |  |  |  |  |  |
|  |  |  |  |  |  |  |  |  |  |  | Suturing | 0.9688 | 1 | 0.9375 |  |  |  |  |  |
|  |  |  |  |  |  |  |  |  |  |  | Peg transfer | 0.875 | 0.9375 | 0.8125 |  |  |  |  |  |
|  |  |  |  |  |  |  |  |  |  |  | Cutting | 0.875 | 0.875 | 0.875 |  |  |  |  |  |
|  |  |  |  |  |  |  |  |  |  |  | Clipping | 0.7333 | 0.6875 | 0.875 |  |  |  |  |  |
| Evaluation of Deep Learning Models for Identifying Surgical Actions and Measuring Performance[21] | Shuja Khalid, Mitchell Goldenberg, Teodor Grantcharov, Babak Taati, Frank Rudzicz | 2020 | Canada | JAMA Network Open | 8 participants | RS | SU, NP, KT | KD (dV) | CNN | N, I, E | Source | Data type | Scheme | Model | Metric | Novice | Intermediate | Expert |  |
|  |  |  |  |  |  |  |  |  |  |  | Embedding analysis | Video | LOSO | bi-LSTM (attention) | Accuracy | 0.77 (0.14) | 0.77 (0.14) | 0.77 (0.14) |  |
|  |  |  |  |  |  |  |  |  |  |  |  |  |  |  | Precision | 0.85 (0.09) | 0.67 (0.07) | 0.79 (0.12) |  |
|  |  |  |  |  |  |  |  |  |  |  |  |  |  |  | Recall | 0.85 (0.05) | 0.69 (0.14) | 0.80 (0.13) |  |
|  |  |  |  |  |  |  |  |  |  |  |  |  |  |  | F1 score | 0.85 (0.07) | 0.68 (0.10) | 0.79 (0.12) |  |
|  |  |  |  |  |  |  |  |  |  |  |  |  | LOUO | Gated recurrent unit | Accuracy | 0.70 (0.21) | 0.70 (0.21) | 0.70 (0.21) |  |
|  |  |  |  |  |  |  |  |  |  |  |  |  |  |  |  |  |  |  |  |
|  |  |  |  |  |  |  |  |  |  |  |  |  |  |  | Precision | 0.91 (0.05) | 0.48 (0.11) | 0.70 (0.15) |  |
|  |  |  |  |  |  |  |  |  |  |  |  |  |  |  | Recall | 0.76 (0.08) | 0.67 (0.19) | 0.75 (0.11) |  |
|  |  |  |  |  |  |  |  |  |  |  |  |  |  |  | F1 score | 0.83 (0.05) | 0.55 (0.12) | 0.72 (0.13) |  |
|  |  |  |  |  |  |  |  |  |  |  | Key point representation analysis | Video | LOUO | Bi-LSTM | Accuracy | 0.73 (0.33) | 0.73 (0.33) | 0.73 (0.33) |  |
|  |  |  |  |  |  |  |  |  |  |  |  |  |  |  | Precision | 1.00 (0) | 0.01 (0) | 1.00 (0) |  |
|  |  |  |  |  |  |  |  |  |  |  |  |  |  |  | Recall | 0.47 (0.18) | 0.29 (0.11) | 1.00 (0) |  |
|  |  |  |  |  |  |  |  |  |  |  |  |  |  |  | F1 score | 0.64 (0.25) | 0.02 (0.01) | 1.00 (0) |  |
| Development and Validation of a 3-Dimensional Convolutional Neural Network for Automatic Surgical Skill Assessment Based on Spatiotemporal Video Analysis[22] | Daichi Kitaguchi, Nobuyoshi Takeshita, Hiroki Matsuzaki, Takahiro Igaki, Hiro Hasegawa, Masaaki Ito | 2021 | Japan | JAMA Network Open | unknown | Lap | Laparoscopic colorectal surgery | VR | CNN | Endoscopic Surgical Skill Qualification System Score | Mean SD accuracy |  | Overall | Medial mobilization | Lateral mobilization | IMA transection | Mesorectal transecion |  |  |
|  |  |  |  |  |  |  |  |  |  |  |  | SD Accuracy | 75.0% (6.3%) | 73.00% | 74.30% | 83.80% | 68.90% |  |  |
| A vision transformer for decoding surgeon activity from surgical videos[23] | Dani Kiyasseh, Runzhuo Ma, Taseen F. Haque, Brian J. Miles, Christian Wagner, Daniel A. Donoho, Animashree Anandkumar & Andrew J. Hung | 2023 | USA | nature biomedical engineering | 42 surgeons | Rob | SU | VR | ML | Low vs. high skill level | AUC (+/-1SD) |  |  |  |  |  |  |  |  |
|  |  |  |  |  |  |  |  |  |  |  | Needle handling | 0.849 (0.06) |  |  |  |  |  |  |  |
|  |  |  |  |  |  |  |  |  |  |  | Needle driving | 0.821 (0.05) |  |  |  |  |  |  |  |
| Sensor-based machine learning for workflow detection and as key to detect expert level in laparoscopic suturing and knot-tying[24] | Karl‑Friedrich Kowalewski, Carly R. Garrow, Mona W. Schmidt, Laura Benner, Beat P. Müller‑Stich, Felix Nickel | 2019 | Germany | Surgical Endoscopy | 28 participants | LS | SU, KT | KD (s): Myoarmband | ML | Group classification (N, I, E) and OSATS score prediction |  | ML algorithm type | Mean Error | r2 |  |  |  |  |  |
|  |  |  |  |  |  |  |  |  |  |  | OSATS Score classification: | Decision forest | 4.45 ± 0.75 | − 0.2 ± 0.69 |  |  |  |  |  |
|  |  |  |  |  |  |  |  |  |  |  |  | Neural networks | 3.71 ± 0.64 | 0.03 ± 0.81 |  |  |  |  |  |
|  |  |  |  |  |  |  |  |  |  |  |  | Boosted decision tree | 4.43 ± 0.60 | 0.19 ± 0.46 |  |  |  |  |  |
|  |  |  |  |  |  |  |  |  |  |  | Group classification | ML algorithm type | Accuracy | Precision | Recall |  |  |  |  |
|  |  |  |  |  |  |  |  |  |  |  |  | Decision jungle | 0.62 | 0.43 | 0.43 |  |  |  |  |
|  |  |  |  |  |  |  |  |  |  |  |  | Neural networks | 0.7 | 0.56 | 0.56 |  |  |  |  |
|  |  |  |  |  |  |  |  |  |  |  |  | Support vector | 0.6 | 0.39 | 0.39 |  |  |  |  |
|  |  |  |  |  |  |  |  |  |  |  |  | Boosted decision tree | 0.66 | 0.56 | 0.56 |  |  |  |  |
| The development of an eye movement‑based deep learning system for laparoscopic surgical skills assessment[25] | R. J. Kuo, Hung‑Jen Chen, Yi‑Hung Kuo | 2022 | Taiwan | Scientific Reports | 10 students | LS | PT | VR, Tobii Pro Nano remote eye tracker data | ML (ensemble learning extreme machine learning (EML), DL (CNN: SVM, RF, extremely randomized trees, MLP = Multilayer Perception, AdaBoost = adaptive boosting, GBDT = Gradient-boosted decision trees, XGBoost = extreme gradient boosting) | N, I, E | Algorithm | Testing accuracy - Mean | STD |  |  |  |  |  |  |
|  |  |  |  |  |  |  |  |  |  |  | SVM | 0.743 | 0.055 |  |  |  |  |  |  |
|  |  |  |  |  |  |  |  |  |  |  | RF | 0.763 | 0.058 |  |  |  |  |  |  |
|  |  |  |  |  |  |  |  |  |  |  | AdaBoost | 0.722 | 0.057 |  |  |  |  |  |  |
|  |  |  |  |  |  |  |  |  |  |  | ETC | 0.762 | 0.06 |  |  |  |  |  |  |
|  |  |  |  |  |  |  |  |  |  |  | MLP | 0.76 | 0.057 |  |  |  |  |  |  |
|  |  |  |  |  |  |  |  |  |  |  | XGB | 0.763 | 0.063 |  |  |  |  |  |  |
|  |  |  |  |  |  |  |  |  |  |  | GB | 0.764 | 0.063 |  |  |  |  |  |  |
|  |  |  |  |  |  |  |  |  |  |  | Ensemble | 0.766 | 0.052 |  |  |  |  |  |  |
|  |  |  |  |  |  |  |  |  |  |  | EML | 0.825 | 0.03 |  |  |  |  |  |  |
| Endoscopic Image-Based Skill Assessment in Robot-Assisted Minimally Invasive Surgery[26] | Gábor Lajkó, Renáta Nagyné Elek, Tamás Haidegger | 2021 | Germany, Hungary, Austria | Sensors (Basel) | 8 participants | RS | SU, NP, KT | KD (dV) | CNN, CNN-LSTM (long short-term memory), ResNET (Residual Neural Network) | binary (N, E) | Efficacy | Suturing task | Needle passing | Knot tying |  |  |  |  |  |
|  |  |  |  |  |  |  |  |  |  |  | CNN | 0.8072 | 0.7966 | 0.8041 |  |  |  |  |  |
|  |  |  |  |  |  |  |  |  |  |  | CNN + LSTM | 0.8158 | 0.8319 | 0.8282 |  |  |  |  |  |
|  |  |  |  |  |  |  |  |  |  |  | ResNet | 0.8189 | 0.8423 | 0.8354 |  |  |  |  |  |
| Deep Learning for Instrument Detection and Assessment of Operative Skill in Surgical Videos[27] | Kyle Lam, Frank P.-W. Lo, Yujian An, Ara Darzi, James M. Kinross, Sanjay Purkayastha, Benny Lo | 2022 | UK | IEEE Transactions on Medical Robotics and Bionics | unknown | Lap | Laparoscopic gastric band insertion | VR | R-CNN | Trainee vs. Expert | Qualitative description |  |  |  |  |  |  |  |  |
| Automation of surgical skill assessment using a three‑stage machine learning algorithm[28] | Joël L. Lavanchy, Joel Zindel, Kadir Kirtac, Isabell Twick, Enes Hosgor, Daniel Candinas & Guido Beldi | 2021 | Switzerland | Scientific Reports | 40 surgeons | Lap | Laparoscopic cholecystectomy | VR | CNN, ML | binary (good vs. Poor surgical skill) and skill level from 1-5 (with +/- 1 deviation) | Accuracy | Good vs. poor surgical skill | Skill level from 1-5 |  |  |  |  |  |  |
|  |  |  |  |  |  |  |  |  |  |  |  | 87 +/- 0.2% | 70 +/- 0.2% |  |  |  |  |  |  |
| Artificial Neural Network for Laparoscopic Skills Classification Using Motion Signals from Apple Watch[29] | Rubbermaid Laverde, Claudia Rueda, Lusvin Amado, David Rojas, Miguel Altuve | 2018 | Colombia | [2018 40th Annual International Conference of the IEEE Engineering in Medicine and Biology Society (EMBC)](https://ieeexplore.ieee.org/xpl/conhome/8471725/proceeding) | 7 volunteers without experience in laparoscopy | LS | PT | KD (s): Apple watch | ANN | N, I, E | Average iteration (of 5) | F1 low | F1 intermediate | F1 high | F1 score |  |  |  |  |
|  |  |  |  |  |  |  |  |  |  |  |  | 85.14% | 91.21% | 81.97% | 86.11% |  |  |  |  |
| Surgeon Technical Skill Assessment using Computer Vision based Analysis[30] | Hei Law, Khurshid Ghani, Jia Deng | 2017 | USA | Proceedings of the 2nd Machine Learning for Healthcare Conference | 12 surgeons | Rob | Robotic prostatectomy | VR | CV, ML (SVM), NN | binary (good vs. poor surgical skill) |  | Accuracy |  |  |  |  |  |  |  |
|  |  |  |  |  |  |  |  |  |  |  | Right hand only | 83.33% |  |  |  |  |  |  |  |
|  |  |  |  |  |  |  |  |  |  |  | Right + left hand annotations | 91.67% |  |  |  |  |  |  |  |
| Automatic assessment of performance in the FLS trainer using computer vision[31] | Aviad Lazar, Gideon Sroka, Shlomi Laufer | 2023 | Israel | Surgical Endoscopy | 27 participants | LS | PT | VR | DL, CV (CNN YOLOv4) | N, I, E | not applicable |  |  |  |  |  |  |  |  |

| Evaluation of Surgical Skills during Robotic Surgery by Deep Learning-Based Multiple Surgical Instrument Tracking in Training and Actual Operations[32] | Dongheon Lee, Hyeong Won Yu, Hyungju Kwon, Hyoun-Joong Kong, Kyu Eun Lee and Hee Chan Kim | 2020 | Korea | Journal of Clinical Medicine | unknown | RS, Rob | Robotic thyroid surgery (DaVinci) performed on patients with thyroid cancer, bilateral axillo-breast approach (BABA) training model | VR | DL, CNN | N, I, E | Accuracy | OSATS | GEARS |  |  |  |  |  |  |
| --- | --- | --- | --- | --- | --- | --- | --- | --- | --- | --- | --- | --- | --- | --- | --- | --- | --- | --- | --- |
|  |  |  |  |  |  |  |  |  |  |  | Linear classifier | 58% | 67% |  |  |  |  |  |  |
|  |  |  |  |  |  |  |  |  |  |  | SVM | 75% | 67% |  |  |  |  |  |  |
|  |  |  |  |  |  |  |  |  |  |  | RF (random forest) | 83% | 83% |  |  |  |  |  |  |
| Clearness of operating field: a surrogate for surgical skills on in vivo clinical data[33] | Daochang Liu, Tingting Jiang, Yizhou Wang, Rulin Miao, Fei Shan, Ziyu Li | 2020 | China | International Journal of Computer Assisted Radiology and Surgery | unknown | Lap | Laparoscopic gastrectomy | VR | NN | modified OTS: 14 skill metrics (1-5pts. Each), OPS: 7 metrics (1-5pts each) |  | Method | % SROCC (Std.) | % PLOCC (Std.) |  |  |  |  |  |
|  |  |  |  |  |  |  |  |  |  |  | OTS =overall technical skills | Direct (automated, L(both)) | 49.4 (0.5) | 53.5 (1.3) |  |  |  |  |  |
|  |  |  |  |  |  |  |  |  |  |  |  | Surrogate (automated, L(both)) | 59.5 (0.4) | 61.5 (0.2) |  |  |  |  |  |
|  |  |  |  |  |  |  |  |  |  |  | OPS = overall procedural skills | Direct (automated, L(both)) | 25.5 (1.3) | 25.0 (1.9) |  |  |  |  |  |
|  |  |  |  |  |  |  |  |  |  |  |  | Surrogate (automated, L(both)) | 41.6 (0.4) | 42.6 (0.5) |  |  |  |  |  |
| Towards Unified Surgical Skill Assessment[34] | Daochang Liu, Qiyue Li, Tingting Jiang, Yizhou Wang, Rulin Miao, Fei Shan, Ziyu Li | 2021 | China | IEEE/CVF Conference on Computer Vision and Pattern Recognition (CVPR) | 8 participants / unknown | RS, Lap | SU, NP, KT / 20 laparoscopic videos of in vivo surgeries for gastric cancer | VR | ML | Modified OSATS score/GRS Score | SROCC |  | Suturing | Needle Passing | Knot Tying | Avg. |  |  |  |
|  |  |  |  |  |  |  |  |  |  |  | Clinical Dataset | 0.565 |  |  |  |  |  |  |  |
|  |  |  |  |  |  |  |  |  |  |  | JIGSAWS DATASET |  | 0.83 | 0.76 | 0.82 | 0.8 |  |  |  |
| An objective approach to evaluate novice robotic surgeons using a combination of kinematics and stepwise cumulative sum (CUSUM) analyses[35] | William B. Lyman, Michael J. Passeri, Keith Murphy, Imran A. Siddiqui, Adeel S. Khan, David A. Iannitti, John B. Martinie, Erin H. Baker, Dionisios Vrochides | 2021 | USA | Surgical Endoscopy | 2 hepato-pancreatobiliary surgery (HPB) fellows | RS | 40 robotic assisted hepaticojejunostomy reconstructions | KD (dV) | ML | binary (N, I) | Accuracy | 89.30% |  |  |  |  |  |  |  |
| Surgical skill levels: Classification and analysis using deep neural network model and motion signals[36] | Xuan Anh Nguyen, Damir Ljuhar, Maurizio Pacilli, Ramesh Mark Nataraja, Sunita Chauhan | 2019 | Australia | Computer Methods and Programs in Biomedicine | 8 participants | RS | SU, NP, KT | KD (dV) | DNN | N, I, E | JIGSAWS DATASET (average classification results %) | Suturing | Needle passing | Knot tying |  |  |  |  |  |
|  |  |  |  |  |  |  |  |  |  |  | CNN-LSTM | 97.2 | 97.3 | 91.5 |  |  |  |  |  |
|  |  |  |  |  |  |  |  |  |  |  | CNN-LSTM + SENET | 98.3 | 97.8 | 94.7 |  |  |  |  |  |
|  |  |  |  |  |  |  |  |  |  |  | CNN-LSTM + SENET + Restart | 98.4 | 98.4 | 94.8 |  |  |  |  |  |
| Ranking surgical skills using an attention-enhanced Siamese network with piecewise aggregated kinematic data[37] | Burçin Buket Oğul, Matthias Gilgien, Suat Özdemir | 2022 | Turkey | International Journal of Computer Assisted Radiology and Surgery | 8 + 12 (JIGSAW + ROSMA) | RS | SU, NP, KT / post and sleeve, pea on a peg and wire chaser | KD (dV) | CNN (Attention-enhanced Siamese Long Short-Term Memory Network) | binary, ternary ranking | Ranking JIGSAWS | Ternary ranking (including skill equivalence) (Accuracy) | Binary ranking (excluding skill equivalence) (Accuracy) |  |  |  |  |  |  |
|  |  |  |  |  |  |  |  |  |  |  | KT | 79.2 | 83.65 |  |  |  |  |  |  |
|  |  |  |  |  |  |  |  |  |  |  | NP | 78.87 | 82.48 |  |  |  |  |  |  |
|  |  |  |  |  |  |  |  |  |  |  | SU | 69.29 | 72.89 |  |  |  |  |  |  |
|  |  |  |  |  |  |  |  |  |  |  | Average | 75.8 | 79.67 |  |  |  |  |  |  |
|  |  |  |  |  |  |  |  |  |  |  | Ranking ROSMA | Accuracy |  |  |  |  |  |  |  |
|  |  |  |  |  |  |  |  |  |  |  | Wire chaser | 75.6 |  |  |  |  |  |  |  |
|  |  |  |  |  |  |  |  |  |  |  | Post and sleeve | 75.1 |  |  |  |  |  |  |  |
|  |  |  |  |  |  |  |  |  |  |  | Pee on a peg | 74.9 |  |  |  |  |  |  |  |
|  |  |  |  |  |  |  |  |  |  |  | Average | 75.2 |  |  |  |  |  |  |  |
|  |  |  |  |  |  |  |  |  |  |  | Regression model (Spearman's Correlation Coefficient) | Present method with actual ranks | Present method with predicted ranks |  |  |  |  |  |  |
|  |  |  |  |  |  |  |  |  |  |  | KT | 0.99 | 0.71 |  |  |  |  |  |  |
|  |  |  |  |  |  |  |  |  |  |  | NP | 0.99 | 0.65 |  |  |  |  |  |  |
|  |  |  |  |  |  |  |  |  |  |  | SU | 0.99 | 0.59 |  |  |  |  |  |  |
|  |  |  |  |  |  |  |  |  |  |  | Performance in individual progress monitoring ROSMA |  |  |  |  |  |  |  |  |
|  |  |  |  |  |  |  |  |  |  |  | Wire chaser | 73.9 |  |  |  |  |  |  |  |
|  |  |  |  |  |  |  |  |  |  |  | Post and sleeve | 66.7 |  |  |  |  |  |  |  |
|  |  |  |  |  |  |  |  |  |  |  | Pee on a peg | 69.4 |  |  |  |  |  |  |  |
|  |  |  |  |  |  |  |  |  |  |  | Average | 70 |  |  |  |  |  |  |  |

| Automatically rating trainee skill at a pediatric laparoscopic suturing task[38] | Yousi A. Oquendo, Elijah W. Riddle, Dennis Hiller, Thane A. Blinman, Katherine J. Kuchenbecker | 2018 | USA | Surgical Endoscopy | 32 participants (med students - fellows) | LS | SU | KD (s): magnetic sensors | ML | OSATS score (summed scores and rounded average scores) | Scoring performance | Summed scores |  |  |  |  |  |  |  |
| --- | --- | --- | --- | --- | --- | --- | --- | --- | --- | --- | --- | --- | --- | --- | --- | --- | --- | --- | --- |
|  |  |  |  |  |  |  |  |  |  |  |  | Plus/ minus 2 accuracy | Plus/ minus 4 accuracy | Correlation |  |  |  |  |  |
|  |  |  |  |  |  |  |  |  |  |  | TMVG (model that used all sensor data streams) | 0.71 | 0.89 | 0.85 |  |  |  |  |  |
| An Automated Skill Assessment Framework Based on Visual Motion Signals and a Deep Neural Network in Robot-Assisted Minimally Invasive Surgery[39] | Mingzhang Pan, Shuo Wang, Jingao Li, Jing Li *1, Xiuze Yang, Ke Liang* | 2023 | China | Sensors | 8 participants | RS | SU | VR | DL (KCF + ResNet) | N, E and N, I, E |  | Accuracy (average) | Precision | Recall | F1-Score |  |  |  |  |
|  |  |  |  |  |  |  |  |  |  |  | Binary (N, E) | 0.92 |  |  |  |  |  |  |  |
|  |  |  |  |  |  |  |  |  |  |  | N |  | 0.99 | 0.91 | 0.95 |  |  |  |  |
|  |  |  |  |  |  |  |  |  |  |  | E |  | 0.92 | 0.93 | 0.93 |  |  |  |  |
|  |  |  |  |  |  |  |  |  |  |  | 3 skill levels | 0.848 |  |  |  |  |  |  |  |
|  |  |  |  |  |  |  |  |  |  |  | N |  | 0.96 | 0.87 | 0.91 |  |  |  |  |
|  |  |  |  |  |  |  |  |  |  |  | I |  | 0.78 | 0.8 | 0.79 |  |  |  |  |
|  |  |  |  |  |  |  |  |  |  |  | E |  | 0.39 | 0.71 | 0.5 |  |  |  |  |
| Objective classification of psychomotor laparoscopic skills of surgeons based on three different approaches[40] | Fernaando Pérez‑Escamirosa, Antonio Alarcón‑Paredes, Gustavo Adolfo Alonso‑Silverio, Ignacio Oropesa, Oscar Camacho‑Nieto, Daniel Lorias‑Espinoza, Arturo Minor‑Martínez | 2019 | Mexico | International Journal of Computer Assisted Radiology and Surgery | 43 participants (med students - experienced surgeons) | LS | PC, PT, SU | VR | ML (neural network based (RBFNet), lazy learner based on distance computation (K-star), tree-based classifier (random forest)) | binary (experienced, non-experienced) | Task | Hold out (75–25) |  |  |  |  |  |  |  |
|  |  |  |  |  |  |  |  |  |  |  |  | Classifier | Accuracy (%) | RMSE | Sensitivity (%) | Specificity (%) | AUC | F1-Score |  |
|  |  |  |  |  |  |  |  |  |  |  | Peg transfer | RBFNets | 81.82 | 0.387 | 81.8 | 72.3 | 0.92 | 0.75 |  |
|  |  |  |  |  |  |  |  |  |  |  |  | K* | 90.91 | 0.305 | 90.9 | 75.8 | 0.92 | 0.8 |  |
|  |  |  |  |  |  |  |  |  |  |  |  | RF | 81.82 | 0.337 | 81.8 | 72.3 | 0.92 | 0.75 |  |
|  |  |  |  |  |  |  |  |  |  |  |  | Avg | 84.85 | 0.343 | 84.83 | 73.46 | 0.92 | 0.77 |  |
|  |  |  |  |  |  |  |  |  |  |  | Pattern cutting | RBFNets | 90.01 | 0.304 | 90.9 | 75.8 | 0.96 | 0.8 |  |
|  |  |  |  |  |  |  |  |  |  |  |  | K* | 98.18 | 0.066 | 100 | 97.55 | 0.99 | 0.92 |  |
|  |  |  |  |  |  |  |  |  |  |  |  | RF | 81.82 | 0.331 | 81.8 | 72.3 | 0.92 | 0.75 |  |
|  |  |  |  |  |  |  |  |  |  |  |  | Avg | 90.61 | 0.317 | 90.9 | 82.7 | 0.96 | 0.82 |  |
|  |  |  |  |  |  |  |  |  |  |  | Intracorporeal knot suture | RBFNets | 90.91 | 0.302 | 90.9 | 75.8 | 0.96 | 0.8 |  |
|  |  |  |  |  |  |  |  |  |  |  |  | K* | 90.91 | 0.302 | 90.9 | 96.6 | 0.96 | 0.86 |  |
|  |  |  |  |  |  |  |  |  |  |  |  | RF | 90.91 | 0.245 | 90.9 | 75.8 | 0.96 | 0.8 |  |
|  |  |  |  |  |  |  |  |  |  |  |  | Avg | 90.91 | 0.283 | 90.9 | 82.73 | 0.96 | 0.82 |  |
|  |  |  |  |  |  |  |  |  |  |  | Task | Leave-one-out cross-validation |  |  |  |  |  |  |  |
|  |  |  |  |  |  |  |  |  |  |  |  | Classifier | Accuracy (%) | RMSE | Sensitivity (%) | Specificity (%) | AUC | F1-Score |  |
|  |  |  |  |  |  |  |  |  |  |  | Peg transfer | RBFNets | 90.7 | 0.239 | 90.7 | 83.2 | 0.94 | 0.8 |  |
|  |  |  |  |  |  |  |  |  |  |  |  | K* | 86.05 | 0.336 | 86 | 74.9 | 0.96 | 0.86 |  |
|  |  |  |  |  |  |  |  |  |  |  |  | RF | 81.4 | 0.324 | 81.4 | 66.5 | 0.9 | 0.76 |  |
|  |  |  |  |  |  |  |  |  |  |  |  | Avg | 86.05 | 0.299 | 86.03 | 74.86 | 0.93 | 0.81 |  |
|  |  |  |  |  |  |  |  |  |  |  | Pattern cutting | RBFNets | 86.05 | 0.329 | 86 | 81.8 | 0.95 | 0.73 |  |
|  |  |  |  |  |  |  |  |  |  |  |  | K* | 90.7 | 0.274 | 90.7 | 97.2 | 0.99 | 0.83 |  |
|  |  |  |  |  |  |  |  |  |  |  |  | RF | 76.74 | 0.341 | 76.7 | 58.1 | 0.89 | 0.67 |  |
|  |  |  |  |  |  |  |  |  |  |  |  | Avg | 84.5 | 0.314 | 84.46 | 79.03 | 0.94 | 0.74 |  |
|  |  |  |  |  |  |  |  |  |  |  | Intracorporeal knot suture | RBFNets | 93.02 | 0.273 | 93 | 77 | 0.9 | 0.86 |  |
|  |  |  |  |  |  |  |  |  |  |  |  | K* | 97.67 | 0.153 | 97.7 | 93 | 0.99 | 0.95 |  |
|  |  |  |  |  |  |  |  |  |  |  |  | RF | 93.02 | 0.197 | 93 | 90.9 | 0.99 | 0.86 |  |
|  |  |  |  |  |  |  |  |  |  |  |  | Avg | 94.57 | 0.207 | 94.56 | 86.96 | 0.96 | 0.89 |  |
| Machine learning-based automatic evaluation of tissue handling skills in laparoscopic colorectal surgery: A retrospective experimental study[41] | Shoma Sasaki, Daichi Kitaguchi, Shin Takenaka, Kei Nakajima, Kimimasa Sasaki, Tateo Ogane, Tateo, Nobuyoshi Takeshita, Naoto, Gotohda, Masaaki Ito | 2022 | Japan | Annals of Surgery | unknown | Lap | Laparoscopic sigmoidectomy | VR | ML | High tissue handling score group, low tissue handling score group, novice surgeon group (classified by means of blood pixels) | Blood pixel count per frame | p-value = 0.267 (no significant correlation between blood pixel count and overall surgical skill score) |  |  |  |  |  |  |  |
| Developing Surgical Skill Level Classification Model Using Visual Metrics and a Gradient Boosting Algorithm[42] | Somayeh B. Shafiei, Saeed Shadpour, James L. Mohler, Kristopher Attwood, Qian Liu, Camille Gutierrez, Mehdi Seilanian Toussi | 2023 | USA | Annals of Surgery open | 11 participants | RS | Blunt dissection, retraction, cold dissection and cutting using scissors, burn dissection (live pigs) | Eye gaze data recorded from TobiiPro2 eyeglasses | ML (Ensemble learning) | Inexperienced, competent, experienced (GEARS Score) | Average % (SD%) | Blunt dissection | Retraction | Cold dissection | Burn dissection |  |  |  |  |
|  |  |  |  |  |  |  |  |  |  |  | Precision | 92 (4) | 96 (1) | 94 (4) | 96 (1) |  |  |  |  |
|  |  |  |  |  |  |  |  |  |  |  | Recall | 95 (4) | 96 (2) | 96 (3) | 96 (2) |  |  |  |  |
|  |  |  |  |  |  |  |  |  |  |  | F1-Score | 93 (4) | 96 (1) | 94 (3) | 96 (2) |  |  |  |  |
|  |  |  |  |  |  |  |  |  |  |  | Accuracy | 95 (3) | 96 (1) | 96 (2) | 96 (2) |  |  |  |  |
| Evaluation of surgical skill using machine learning with optimal wearable sensor locations[43] | Rahul Soangra, R. Sivakumar, E. R. Anirudh, Sai Viswanth Reddy Y., Emmanuel B. John | 2022 | USA | PLOS ONE | 26 surgeons | LS, RS | PT, KT | KD (s): wireless wearable sensors (surface EMGs, accelerometers) | ML (RF classifier, SVM, Naïve Bayes classifier) | N, I, E | Performance of a combination of muscles (3 classification model) |  |  |  |  |  |  |  |  |
|  |  |  |  |  |  |  |  |  |  |  |  | Deltoid, ECU (extensor carpi ulnaris), and biceps |  |  |  | ECU and deltoid |  |  |  |
|  |  |  |  |  |  |  |  |  |  |  | Algorithm | Accuracy | Precision | Recall | F1 score | Accuracy | Precision | Recall | F1 score |
|  |  |  |  |  |  |  |  |  |  |  | Random Forest | 0.52 | 0.54 | 0.46 | 0.45 | 0.58 | 0.46 | 0.51 | 0.51 |
|  |  |  |  |  |  |  |  |  |  |  | SVM | 0.5 | 0.66 | 0.43 | 0.41 | 0.5 | 0.33 | 0.4 | 0.36 |
|  |  |  |  |  |  |  |  |  |  |  | Naive Bayes | 0.41 | 0.43 | 0.43 | 0.43 | 0.29 | 0.33 | 0.33 | 0.29 |
|  |  |  |  |  |  |  |  |  |  |  |  | ECU and biceps |  |  |  |  |  |  |  |
|  |  |  |  |  |  |  |  |  |  |  |  | Accuracy | Precision | Recall | F1 score |  |  |  |  |
|  |  |  |  |  |  |  |  |  |  |  | Random Forest | 0.53 | 0.47 | 0.45 | 0.28 |  |  |  |  |
|  |  |  |  |  |  |  |  |  |  |  | SVM | 0.45 | 0.47 | 0.39 | 0.37 |  |  |  |  |
|  |  |  |  |  |  |  |  |  |  |  | Naive Bayes | 0.44 | 0.43 | 0.39 | 0.39 |  |  |  |  |
| Surgical Skill Evaluation From Robot-Assisted Surgery Recordings[44] | Abed Soleymani, Ali Akbar Sadat Asl, Mojtaba Yeganejou, Scott Dick, Mahdi Tavakoli, Xingyu Li | 2021 | Canada | 2021 International Symposium on Medical Robotics (ISMR) | 8 participants | RS | SU, NP, KT | VR | DL | N, I, E | Accuracy | 97.27 +/-2.35 |  |  |  |  |  |  |  |
| A Domain-Adapted Machine Learning Approach for Visual Evaluation and Interpretation of Robot-Assisted Surgery Skills[45] | Abed Soleymani, Xingyu Li, Mahdi Tavakoli | 2022 | Canada | IEEE Robotics and Automation Letters | 8 participants | RS | SU, NP, KT | KD (dV) | ML (SVM) | Qualitative description | not applicable |  |  |  |  |  |  |  |  |
| Feasibility of an AI-Based Measure of the Hand Motions of Expert and Novice Surgeons[46] | Munenori Uemura, Morimasa Tomikawa, Tiejun Miao, Ryota Souzaki, Satoshi Ieiri, Tomohiko Akahoshi, Alan K. Lefor, Makoto Hashizume | 2018 | Japan | Computational and Mathematical Methods in Medicine | 67 surgeons | LS | SU | KD (s): magnetic sensors | NN | Novice, Expert | correctly distinguished | 79% of the participants |  |  |  |  |  |  |  |
| Evaluating robotic-assisted surgery training videos with multi-task convolutional neural networks[47] | Yihao Wang, Jessica Dai, Tara N. Morgan, Mohamed Elsaied, Alaina Garbens, Xingming Qu, Ryan Steinberg, Jeffrey Gahan, Eric C. Larson | 2021 | USA | Journal of Robotic Surgery | 18 surgeons | RS | SU (artificial urinary tissue in the final step of a prostatectomy) | VR | CNN | GEARS scores (1-5)// expert >25, intermediate 20–25, and novice <20. | GEARS Score +/- 1 point | 86.10% |  |  |  |  |  |  |  |
|  |  |  |  |  |  |  |  |  |  |  | GEARS Score +/- 2 points | 1 |  |  |  |  |  |  |  |
|  |  |  |  |  |  |  |  |  |  |  |  | Depth perception | Bi-manual dexterity | Efficiency | Force sens. | Autonomy | Robotic control |  |  |
|  |  |  |  |  |  |  |  |  |  |  | Matching | 94.40% | 77.80% | 94.40% | 83.30% | 72.20% | 88.90% |  |  |
|  |  |  |  |  |  |  |  |  |  |  | Expert vs. Intermediate vs. Novice |  |  |  |  |  |  |  |  |
|  |  |  |  |  |  |  |  |  |  |  | Classified correctly | 83.50% |  |  |  |  |  |  |  |
|  |  |  |  |  |  |  |  |  |  |  | Novice | 100% (11/11) |  |  |  |  |  |  |  |
|  |  |  |  |  |  |  |  |  |  |  | Intermediate | 80% (4/5) |  |  |  |  |  |  |  |
|  |  |  |  |  |  |  |  |  |  |  | Expert | 0% (0/2) |  |  |  |  |  |  |  |
| Deep learning with convolutional neural network for objective skill evaluation in robot-assisted surgery[48] | Ziheng Wang, Ann Majewicz Fey | 2018 | USA | International Journal of Computer Assisted Radiology and Surgery | 8 participants | RS | SU, NP, KT | KD (dV) | DL, CNN | N, I, E (self-proclaimed > practice hours vs. GRS-based) | Accuracy | Self-proclaimed | GRS based |  |  |  |  |  |  |
|  |  |  |  |  |  |  |  |  |  |  | Suturing | 93.4 | 92.5 |  |  |  |  |  |  |
|  |  |  |  |  |  |  |  |  |  |  | Needle passing | 89.8 | 95.4 |  |  |  |  |  |  |
|  |  |  |  |  |  |  |  |  |  |  | Knot tying | 84.9 | 91.3 |  |  |  |  |  |  |
| SATR-DL: Improving Surgical Skill Assessment and Task Recognition in Robot-assisted Surgery with Deep Neural Networks[49] | Ziheng Wang, Ann Majewicz Fey | 2018 | USA | 2018 40th Annual International Conference of the IEEE Engineering in Medicine and Biology Society (EMBC) | 8 participants | RS | SU, NP, KT | KD (dV) | DNN | N, I, E |  | Interval-level classification |  |  |  | Trial-level classification |  |  |  |
|  |  |  |  |  |  |  |  |  |  |  |  | Precision | Recall | F1-score | Overall Accuracy | Precision | Recall | F1-score | Overall Accuracy |
|  |  |  |  |  |  |  |  |  |  |  | Novice | 0.94 | 0.96 | 0.95 | 0.92 | 0.95 | 0.98 | 0.97 | 0.966 |
|  |  |  |  |  |  |  |  |  |  |  | Intermediate | 0.88 | 0.77 | 0.82 |  | 1 | 0.9 | 0.95 |  |
|  |  |  |  |  |  |  |  |  |  |  | Expert | 0.9 | 0.95 | 0.93 |  | 0.97 | 1 | 0.98 |  |
| Automated surgical skill assessment in RMIS training[50] | Aneeq Zia, Irfan Essa | 2018 | USA | International Journal of Computer Assisted Radiology and Surgery | 8 participants | RS | SU, NP, KT | KD (dV) | ML (Support vector regression) | N, I, E and modified OSATS/GRS scores | Self-proclaimed skill classification results | Suturing |  | Knot tying |  | Needle passing |  |  |  |
|  |  |  |  |  |  |  |  |  |  |  |  | LOSO | LOUO | LOSO | LOUO | LOSO | LOUO |  |  |
|  |  |  |  |  |  |  |  |  |  |  | SMT | 99 | 35.3 | 99.6 | 32.3 | 99.9 | 57.1 |  |  |
|  |  |  |  |  |  |  |  |  |  |  | DCT | 100 | 64.7 | 99.7 | 54.8 | 99.9 | 35.7 |  |  |
|  |  |  |  |  |  |  |  |  |  |  | DFT | 100 | 64.7 | 99.9 | 51.6 | 99.9 | 46.4 |  |  |
|  |  |  |  |  |  |  |  |  |  |  | ApEn | 100 | 88.2 | 99.9 | 77.4 | 100 | 85.7 |  |  |
|  |  |  |  |  |  |  |  |  |  |  | Modified-OSATS scores / GRS prediction results |  |  |  |  |  |  |  |  |
|  |  |  |  |  |  |  |  |  |  |  | Spearman's correlation coefficient, “*” means a p-value < 0.05 for the corresponding ρ | Suturing |  | Knot tying |  | Needle passing |  |  |  |
|  |  |  |  |  |  |  |  |  |  |  |  | LOSO | LOUO | LOSO | LOUO | LOSO | LOUO |  |  |
|  |  |  |  |  |  |  |  |  |  |  | SMT | 0.25 \| 0.46* | −0.08 \| −0.28 | 0.41* \| 0.39* | 0.18 \| 0.21 | −0.12 \| 0.09 | 0.07 \| −0.60* |  |  |
|  |  |  |  |  |  |  |  |  |  |  | DCT | 0.57* \| 0.68* | 0.10 \| 0.08 | 0.59* \| 0.76* | 0.49 \| 0.73* | 0.22 \| 0.26* | −0.16 \| 0.09 |  |  |
|  |  |  |  |  |  |  |  |  |  |  | DFT | 0.45* \| 0.49* | −0.28 \| −0.29 | 0.31 \| 0.32* | 0.46* \| 0.47* | 0.44* \| 0.53* | 0.37 \| 0.19 |  |  |
|  |  |  |  |  |  |  |  |  |  |  | ApEn | 0.31* \| 0.49* | 0.43 \| 0.40* | 0.26 \| 0.14* | 0.02 \| 0.12 | 0.16 \| 0.06 | 0.21 \| −0.21 |  |  |
|  |  |  |  |  |  |  |  |  |  |  | SMT+DCT | 0.48* \| 0.61* | 0.01 \| 0.01 | 0.66*\| 0.71* | 0.46 \| 0.78* | 0.14 \| −0.16 | −0.23 \| −0.14 |  |  |
|  |  |  |  |  |  |  |  |  |  |  | SMT+DFT | 0.40* \| 0.60* | −0.21 \| −0.49* | 0.36 \| 0.39* | 0.52* \| 0.48* | 0.39* \| 0.54* | 0.33 \| 0.13 |  |  |
|  |  |  |  |  |  |  |  |  |  |  | SMT+ApEn | 0.28* \| 0.35* | 0.41 \| 0.42* | 0.18 \| 0.36* | 0.06 \| 0.12 | 0.12 \| −0.06 | 0.15 \| −0.29 |  |  |
|  |  |  |  |  |  |  |  |  |  |  | SMT+DCT+DFT | 0.57* \| 0.64* | 0.16 \| 0.10 | 0.58* \| 0.70* | 0.56*\| 0.73* | 0.36* \| 0.38* | 0.50* \| 0.23 |  |  |
|  |  |  |  |  |  |  |  |  |  |  | DCT+DFT | 0.56* \| 0.66* | 0.13 \| 0.14 | 0.53* \| 0.68* | 0.55* \| 0.73* | 0.41* \| 0.47* | 0.53* \| 0.28 |  |  |
|  |  |  |  |  |  |  |  |  |  |  | DCT+DFT+ApEn | 0.59* \| 0.75* | 0.43* \| 0.37* | 0.57* \| 0.63* | 0.48 \| 0.60* | 0.37 \| 0.46* | 0.23 \| 0.25 |  |  |
|  |  |  |  |  |  |  |  |  |  |  | SMT+DCT+DFT+ApEn | 0.47* \| 0.66* | 0.45*\| 0.37* | 0.55* \| 0.61* | 0.49 \| 0.62* | 0.45*\| 0.45* | −0.21 \| −0.19 |  |  |

Abbreviations: LS = laparoscopic simulator, Lap = laparoscopic surgery, RS = robotic simulator, Rob = robotic surgery, PC = pattern cutting, SU = suturing, NP = needle-passing, KT = knot-tying, PT = peg transfer, VR = video recordings, KD (dv) = kinematic data collected by daVinci systems, fNIRS = functional near-infrared spectroscopy, KD (s) = kinematic data collected by external sensors, ANN = artificial neural network, CV = computer vision, CNN = convolutional neural network, DL = deep learning, ML = machine learning, N = novice, I = intermediate, E = expert, PLACE = Pelvic Lymphadenectomy Assessment and Completion Evaluation, GEARS = Global Evaluative Assessments of Robotic Skills, FLS = Fundamentals of Laparoscopic Surgery, ESSQS = Endoscopic Surgical Skill Qualification System, OSATS = Objective Structured Assessment of Technical Skills

Table S4: Risk of bias assessment of included studies

| **Study** | **Population** | **Index test** | **Reference standard** | **Flow and timing** | **Data management** |
| --- | --- | --- | --- | --- | --- |
|  | Was the study population described? | Was cross-validation or external validation performed? | Who performed the annotation? Performance validation? | Did all participants receive the same reference standard? Were all patients included in the analysis | Was ethical approval specified? |
| Alonso-Silverio et al.[1] | ✓ | ✓ | 🗶 | ✓ | 🗶 |
| Anastasiou et al.[2] | ✓ | ✓ | 🗶 | ✓ | 🗶 |
| Anh et al.[3] | ✓ | 🗶 | 🗶 | ✓ | 🗶 |
| Baghdadi et al.[4] | 🗶 | 🗶 | 🗶 | ✓ | 🗶 |
| Benmansour et al.[5] | 🗶 | 🗶 | 🗶 | 🗶 | 🗶 |
| Benmansour et al.[6] | 🗶 | ✓ | 🗶 | 🗶 | 🗶 |
| Brown et al. [7] | ✓ | ✓ | ✓ | ✓ | ✓ |
| Castro et al.[8] | ✓ | ✓ | 🗶 | ✓ | 🗶 |
| Fard et al.[9] | 🗶 | ✓ | 🗶 | ✓ | 🗶 |
| Fathabadi et al.[10] | 🗶 | ✓ | 🗶 | 🗶 | 🗶 |
| Fawaz et al.[11] | ✓ | ✓ | 🗶 | ✓ | 🗶 |
| Forestier et al.[12] | ✓ | ✓ | 🗶 | ✓ | 🗶 |
| French et al.[13] | ✓ | ✓ | 🗶 | 🗶 | ✓ |
| Funke et al.[14] | ✓ | ✓ | 🗶 | ✓ | ✓ |
| Gao et al.[15] | ✓ | ✓ | 🗶 | ✓ | ✓ |
| Islam et al.[16] | 🗶 | 🗶 | ✓ | ✓ | 🗶 |
| Jin et al.[17] | 🗶 | 🗶 | ✓ | ✓ | 🗶 |
| Juarez-Villalobos et al.[18] | ✓ | ✓ | 🗶 | ✓ | 🗶 |
| Keles et al.[19] | ✓ | ✓ | 🗶 | ✓ | ✓ |
| Kelly et al.[20] | ✓ | ✓ | ✓ | ✓ | ✓ |
| Khalid et al.[21] | ✓ | ✓ | 🗶 | ✓ | ✓ |
| Kitaguchi et al.[22] | ✓ | ✓ | ✓ | ✓ | ✓ |
| Kiyasseh et al.[23] | ✓ | ✓ | ✓ | ✓ | ✓ |
| Kowalewski et al.[24] | ✓ | ✓ | ✓ | ✓ | ✓ |
| Kuo et al.[25] | ✓ | ✓ | 🗶 | ✓ | ✓ |
| Lajkó et al.[26] | 🗶 | ✓ | 🗶 | ✓ | 🗶 |
| Lam et al.[27] | 🗶 | 🗶 | ✓ | ✓ | 🗶 |
| Lavanchy et al.[28] | ✓ | ✓ | ✓ | ✓ | ✓ |
| Laverde et al.[29] | ✓ | ✓ | 🗶 | ✓ | ✓ |
| Law et al.[30] | ✓ | ✓ | ✓ | ✓ | 🗶 |
| Lazar et al.[31] | ✓ | 🗶 | 🗶 | ✓ | ✓ |
| Lee et al.[32] | 🗶 | ✓ | 🗶 | ✓ | 🗶 |
| Liu et al.[33] | ✓ | 🗶 | ✓ | ✓ | ✓ |
| Liu et al.[34] | ✓ | ✓ | ✓ | ✓ | 🗶 |
| Lyman et al.[35] | ✓ | 🗶 | 🗶 | 🗶 | 🗶 |
| Nguyen et al.[36] | ✓ | ✓ | 🗶 | ✓ | 🗶 |
| Oğul et al.[37] | 🗶 | ✓ | 🗶 | ✓ | ✓ |
| Oquendo et al.[38] | ✓ | ✓ | ✓ | ✓ | ✓ |
| Pan et al.[39] | ✓ | ✓ | ✓ | ✓ | 🗶 |
| Pérez-Escamirosa et al.[40] | ✓ | ✓ | 🗶 | ✓ | ✓ |
| Sasaki et al.[41] | ✓ | 🗶 | ✓ | ✓ | ✓ |
| Shafiei et al.[42] | ✓ | ✓ | ✓ | ✓ | ✓ |
| Soangra et al.[43] | ✓ | 🗶 | 🗶 | ✓ | ✓ |
| Soleymani et al.[44] | ✓ | ✓ | 🗶 | ✓ | 🗶 |
| Soleymani et al.[45] | ✓ | ✓ | 🗶 | ✓ | ✓ |
| Uemura et al.[46] | ✓ | ✓ | 🗶 | 🗶 | 🗶 |
| Wang Y. et al.[47] | 🗶 | ✓ | 🗶 | ✓ | ✓ |
| Wang Z. et al.[48] | ✓ | ✓ | 🗶 | ✓ | ✓ |
| Wang Z. et al.[49] | 🗶 | ✓ | 🗶 | ✓ | 🗶 |
| Zia et al.[50] | ✓ | ✓ | 🗶 | ✓ | ✓ |

✓ = low risk of bias, 🗶 = high risk of bias

References

1. Alonso-Silverio GA, Perez-Escamirosa F, Bruno-Sanchez R, Ortiz-Simon JL, Munoz-Guerrero R, Minor-Martinez A, Alarcon-Paredes A (2018) Development of a Laparoscopic Box Trainer Based on Open Source Hardware and Artificial Intelligence for Objective Assessment of Surgical Psychomotor Skills. Surg Innov 25:380–388. https://doi.org/10.1177/1553350618777045

2. Anastasiou D, Jin Y, Stoyanov D, Mazomenos E (2023) Keep Your Eye on the Best: Contrastive Regression Transformer for Skill Assessment in Robotic Surgery. IEEE Robot Autom Lett 8:1755–1762. https://doi.org/10.1109/LRA.2023.3242466

3. Anh NX, Chauhan S, Nataraja RM (2020) Towards near real-time assessment of surgical skills: A comparison of feature extraction techniques. Comput Methods Programs Biomed 187:105234. https://doi.org/10.1016/j.cmpb.2019.105234

4. Baghdadi A, Hussein AA, Ahmed Y, Guru KA, Cavuoto LA (2019) A computer vision technique for automated assessment of surgical performance using surgeons’ console-feed videos. Int J Comput Assist Radiol Surg 14:697–707. https://doi.org/10.1007/s11548-018-1881-9

5. Benmansour M, Handouzi W, Malti A (2018) A neural network architecture for automatic and objective surgical skill assessment. In Proceedings 2018 3^rd^ International Conference on Electrical Sciences and Technologies in Maghreb (CISTEM). https://doi.org/10.1109/CISTEM.2018.8613550

6. Benmansour M, Malti A, Jannin P (2023) Deep neural network architecture for automated soft surgical skills evaluation using objective structured assessment of technical skills criteria. Int J Comput Assist Radiol Surg 18:929–937. https://doi.org/10.1007/s11548-022-02827-5

7. Brown JD, O Brien CE, Leung SC, Dumon KR, Lee DI, Kuchenbecker KJ (2017) Using Contact Forces and Robot Arm Accelerations to Automatically Rate Surgeon Skill at Peg Transfer. IEEE Trans Biomed Eng 64:2263–2275. https://doi.org/10.1109/TBME.2016.2634861

8. Castro D, Pereira D, Zanchettin C, Macêdo D, Bezerra BLD (2019) Towards Optimizing Convolutional Neural Networks for Robotic Surgery Skill Evaluation. In Proceedings 2019 International Joint Conference on Neural Networks (IJCNN). https://doi.org/10.1109/IJCNN.2019.8852341

9. Fard MJ, Ameri S, Darin Ellis R, Chinnam RB, Pandya AK, Klein MD (2018) Automated robot-assisted surgical skill evaluation: Predictive analytics approach. Int J Med Robot 14:e1850. https://doi.org/10.1002/rcs.1850

10. Fathabadi FR, Grantner JL, Shebrain SA, Abdel-Qader I (2021) Surgical Skill Assessment System Using Fuzzy Logic in a Multi-Class Detection of Laparoscopic Box-Trainer Instruments. In Proceedings 2021 IEEE International Conference on Systems, Man, and Cybernetics (SMC). https://doi.org/10.1109/SMC52423.2021.9658766

11. Fawaz HI, Forestier G, Weber J, Idoumghar L, Muller PA (2019) Accurate and interpretable evaluation of surgical skills from kinematic data using fully convolutional neural networks. Int J Comput Assist Radiol Surg 14:1611–1617. https://doi.org/10.1007/s11548-019-02039-4

12. Forestier G, Petitjean F, Senin P, Despinoy F, Huaulme A, Fawaz HI, Weber J, Idoumghar L, Muller PA, Jannin P (2018) Surgical motion analysis using discriminative interpretable patterns. Artif Intell Med 91:3–11. https://doi.org/10.1016/j.artmed.2018.08.002

13. French A, Kowalewski TM, Lendvay TS, Sweet RM (2017) Predicting surgical skill from the first N seconds of a task: value over task time using the isogony principle. Int J Comput Assist Radiol Surg 12:1161–1170. https://doi.org/10.1007/s11548-017-1606-5

14. Funke I, Speidel S, Mees ST, Weitz J (2019) Video-based surgical skill assessment using 3D convolutional neural networks. Int J Comput Assist Radiol Surg 14:1217–1225. https://doi.org/10.1007/s11548-019-01995-1

15. Gao Y, Yan P, Kruger U, Cavuoto L, Schwaitzberg S, De S, Intes X (2021) Functional Brain Imaging Reliably Predicts Bimanual Motor Skill Performance in a Standardized Surgical Task. IEEE Trans Biomed Eng 68:2058–2066. https://doi.org/10.1109/TBME.2020.3014299

16. Islam G, Kahol K, Li BX, Smith M, Patel VL (2016) Affordable, web-based surgical skill training and evaluation tool. J Biomed Inform 59:102–114. https://doi.org/10.1016/j.jbi.2015.11.002

17. Jin A, Yeung S, Jopling J, Krause J, Azagury D, Milstein A, Fei-Fei L (2018) Tool Detection and Operative Skill Assessment in Surgical Videos Using Region-Based Convolutional Neural Networks. In Proceedings 2018 IEEE Winter Conference on Applications of Computer Vision (WACV). https://doi.org/10.1109/WACV.2018.00081

18. Juarez-Villalobos L, Hevia-Montiel N, Perez-Gonzalez J (2021) Machine Learning based Classification of Local Robotic Surgical Skills in a Training Tasks Set. Annu Int Conf IEEE Eng Med Biol Soc IEEE Eng Med Biol Soc Annu Int Conf 2021:. https://doi.org/10.1109/EMBC46164.2021.9629579

19. Keles HO, Cengiz C, Demiral I, Ozmen MM, Omurtag A (2021) High density optical neuroimaging predicts surgeons’s subjective experience and skill levels. PLoS ONE 16:e0247117. https://doi.org/10.1371/journal.pone.0247117

20. Kelly JD, Kowalewski TM, Petersen A, Lendvay TS (2020) Bidirectional long short-term memory for surgical skill classification of temporally segmented tasks. Int J Comput Assist Radiol Surg 15:2079–2088. https://doi.org/10.1007/s11548-020-02269-x

21. Khalid S, Goldenberg M, Grantcharov T, Taati B, Rudzicz F (2020) Evaluation of Deep Learning Models for Identifying Surgical Actions and Measuring Performance. JAMA Netw OPEN 3:. https://doi.org/10.1001/jamanetworkopen.2020.1664

22. Kitaguchi D, Takeshita N, Matsuzaki H, Igaki T, Hasegawa H, Ito M (2021) Development and Validation of a 3-Dimensional Convolutional Neural Network for Automatic Surgical Skill Assessment Based on Spatiotemporal Video Analysis. JAMA Netw Open 4:e2120786. https://doi.org/10.1001/jamanetworkopen.2021.20786

23. Kiyasseh D, Ma R, Haque TF, Miles BJ, Wagner C, Donoho DA, Anandkumar A, Hung AJ (2023) A vision transformer for decoding surgeon activity from surgical videos. Nat Biomed Eng. https://doi.org/10.1038/s41551-023-01010-8

24. Kowalewski KF, Garrow CR, Schmidt MW, Benner L, Muller-Stich BP, Nickel F (2019) Sensor-based machine learning for workflow detection and as key to detect expert level in laparoscopic suturing and knot-tying. Surg Endosc Interv Tech 33:3732–3740. https://doi.org/10.1007/s00464-019-06667-4

25. Kuo RJ, Chen H-J, Kuo Y-H (2022) The development of an eye movement-based deep learning system for laparoscopic surgical skills assessment. Sci Rep 12:11036. https://doi.org/10.1038/s41598-022-15053-5

26. Lajko G, Nagyne Elek R, Haidegger T (2021) Endoscopic Image-Based Skill Assessment in Robot-Assisted Minimally Invasive Surgery. Sensors 21:. https://doi.org/10.3390/s21165412

27. Lam K, Lo FP-W, An Y, Darzi A, Kinross JM, Purkayastha S, Lo B (2022) Deep Learning for Instrument Detection and Assessment of Operative Skill in Surgical Videos. IEEE Trans Med Robot Bionics 4:1068–1071. https://doi.org/10.1109/TMRB.2022.3214377

28. Lavanchy JL, Zindel J, Kirtac K, Twick I, Hosgor E, Candinas D, Beldi G (2021) Automation of surgical skill assessment using a three-stage machine learning algorithm. Sci Rep 11:5197. https://doi.org/10.1038/s41598-021-84295-6

29. Laverde R, Rueda C, Amado L, Rojas D, Altuve M (2018) Artificial Neural Network for Laparoscopic Skills Classification Using Motion Signals from Apple Watch. In Proceedings 2018 40th Annual International Conference of the IEEE Engineering in Medicine and Biology Society (EMBC). https://doi.org/10.1109/EMBC.2018.8513561

30. Law H, Zhang Y, Kim T-K, Miller D, Montie J, Deng J, Ghani K (2018) Surgeon technical skill assessment using computer vision-based analysis. J Urol 199:e1138

31. Lazar A, Sroka G, Laufer S (2023) Automatic assessment of performance in the FLS trainer using computer vision. Surg Endosc. https://doi.org/10.1007/s00464-023-10132-8

32. Lee D, Yu HW, Kwon H, Kong H-J, Lee KE, Kim HC (2020) Evaluation of surgical skills during robotic surgery by deep learning-based multiple surgical instrument tracking in training and actual operations. J Clin Med 9:1–15. https://doi.org/10.3390/jcm9061964

33. Liu D, Jiang T, Wang Y, Miao R, Shan F, Li Z (2020) Clearness of operating field: a surrogate for surgical skills on in vivo clinical data. Int J Comput Assist Radiol Surg 15:1817–1824. https://doi.org/10.1007/s11548-020-02267-z

34. Liu D, Li Q, Jiang T, Wang Y, Miao R, Shan F, Li Z (2021) Towards Unified Surgical Skill Assessment. In Proceedings 2021 IEEE/CVF Conference on Computer Vision and Pattern Recognition (CVPR). https://doi.org/10.1109/CVPR46437.2021.00940

35. Lyman WB, Passeri MJ, Murphy K, Iannitti DA, Martinie JB, Baker EH, Vrochides D, Siddiqui IA, Khan AS (2021) An objective approach to evaluate novice robotic surgeons using a combination of kinematics and stepwise cumulative sum (CUSUM) analyses. Surg Endosc 35:2765–2772. https://doi.org/10.1007/s00464-020-07708-z

36. Nguyen XA, Ljuhar D, Pacilli M, Nataraja RM, Chauhan S (2019) Surgical skill levels: Classification and analysis using deep neural network model and motion signals. Comput METHODS PROGRAMS Biomed 177:1–8. https://doi.org/10.1016/j.cmpb.2019.05.008

37. Oğul BB, Gilgien M, Özdemir S (2022) Ranking surgical skills using an attention-enhanced Siamese network with piecewise aggregated kinematic data. Int J Comput Assist Radiol Surg 17:1039–1048. https://doi.org/10.1007/s11548-022-02581-8

38. Oquendo YA, Riddle EW, Hiller D, Blinman TA, Kuchenbecker KJ (2018) Automatically rating trainee skill at a pediatric laparoscopic suturing task. Surg Endosc Interv Tech 32:1840–1857. https://doi.org/10.1007/s00464-017-5873-6

39. Pan M, Wang S, Li J, Li J, Yang X, Liang K (2023) An Automated Skill Assessment Framework Based on Visual Motion Signals and a Deep Neural Network in Robot-Assisted Minimally Invasive Surgery. Sensors 23:4496. https://doi.org/10.3390/s23094496

40. Perez-Escamirosa F, Alarcon-Paredes A, Alonso-Silverio GA, Oropesa I, Camacho-Nieto O, Lorias-Espinoza D, Minor-Martinez A (2020) Objective classification of psychomotor laparoscopic skills of surgeons based on three different approaches. Int J Comput Assist Radiol Surg 15:27–40. https://doi.org/10.1007/s11548-019-02073-2

41. Sasaki S, Kitaguchi D, Takenaka S, Nakajima K, Sasaki K, Ogane T, Takeshita N, Gotohda N, Ito M (2022) Machine learning-based Automatic Evaluation of Tissue Handling Skills in Laparoscopic Colorectal Surgery: A Retrospective Experimental Study. Ann Surg https://doi.org/10.1097/SLA.0000000000005731

42. Shafiei SB, Shadpour S, Mohler JL, Attwood K, Liu Q, Gutierrez C, Toussi MS (2023) Developing Surgical Skill Level Classification Model Using Visual Metrics and a Gradient Boosting Algorithm. Ann Surg Open 4:e292. https://doi.org/10.1097/AS9.0000000000000292

43. Soangra R, Sivakumar R, Anirudh ER, Reddy Y. SV, John EB (2022) Evaluation of surgical skill using machine learning with optimal wearable sensor locations. PLOS ONE 17:e0267936. https://doi.org/10.1371/journal.pone.0267936

44. Soleymani A, Asl AAS, Yeganejou M, Dick S, Tavakoli M, Li X (2021) Surgical Skill Evaluation From Robot-Assisted Surgery Recordings. In Proceedings 2021 International Symposium on Medical Robotics (ISMR). https://doi.org/10.1109/ISMR48346.2021.9661527

45. Soleymani A, Li X, Tavakoli M (2022) A Domain-Adapted Machine Learning Approach for Visual Evaluation and Interpretation of Robot-Assisted Surgery Skills. IEEE Robot Autom Lett 7:8202–8208. https://doi.org/10.1109/LRA.2022.3186769

46. Uemura M, Tomikawa M, Akahoshi T, Lefor AK, Hashizume M, Miao T, Souzaki R, Ieiri S (2018) Feasibility of an AI-Based Measure of the Hand Motions of Expert and Novice Surgeons. Comput Math Methods Med 2018:9873273. https://doi.org/10.1155/2018/9873273

47. Wang Y, Dai J, Morgan TN, Elsaied M, Garbens A, Qu X, Steinberg R, Gahan J, Larson EC (2021) Evaluating robotic-assisted surgery training videos with multi-task convolutional neural networks. J Robot Surg. https://doi.org/10.1007/s11701-021-01316-2

48. Wang ZH, Fey AM (2018) Deep learning with convolutional neural network for objective skill evaluation in robot-assisted surgery. Int J Comput Assist Radiol Surg 13:1959–1970. https://doi.org/10.1007/s11548-018-1860-1

49. Wang Z, Fey AM (2018) SATR-DL: Improving Surgical Skill Assessment And Task Recognition In Robot-Assisted Surgery With Deep Neural Networks. Annu Int Conf IEEE Eng Med Biol Soc IEEE Eng Med Biol Soc Annu Int Conf 2018:1793–1796. https://doi.org/10.1109/EMBC.2018.8512575

50. Zia A, Essa I (2018) Automated surgical skill assessment in RMIS training. Int J Comput Assist Radiol Surg 13:731–739. https://doi.org/10.1007/s11548-018-1735-5
